# Hybrid stochastic simplifications for multiscale gene networks

**DOI:** 10.1186/1752-0509-3-89

**Published:** 2009-09-07

**Authors:** Alina Crudu, Arnaud Debussche, Ovidiu Radulescu

**Affiliations:** 1 IRMAR UMR CNRS 6625 Université de Rennes1, Campus de Beaulieu, 35042 Rennes, France; 2 ENS Cachan, Antenne de Bretagne Avenue Robert Schumann, 35170, BRUZ, France; 3 INRIA-IRISA, Campus de Beaulieu, 35042 Rennes, France

## Abstract

**Background:**

Stochastic simulation of gene networks by Markov processes has important applications in molecular biology. The complexity of exact simulation algorithms scales with the number of discrete jumps to be performed. Approximate schemes reduce the computational time by reducing the number of simulated discrete events. Also, answering important questions about the relation between network topology and intrinsic noise generation and propagation should be based on general mathematical results. These general results are difficult to obtain for exact models.

**Results:**

We propose a unified framework for hybrid simplifications of Markov models of multiscale stochastic gene networks dynamics. We discuss several possible hybrid simplifications, and provide algorithms to obtain them from pure jump processes. In hybrid simplifications, some components are discrete and evolve by jumps, while other components are continuous. Hybrid simplifications are obtained by partial Kramers-Moyal expansion [1-3] which is equivalent to the application of the central limit theorem to a sub-model. By averaging and variable aggregation we drastically reduce simulation time and eliminate non-critical reactions. Hybrid and averaged simplifications can be used for more effective simulation algorithms and for obtaining general design principles relating noise to topology and time scales. The simplified models reproduce with good accuracy the stochastic properties of the gene networks, including waiting times in intermittence phenomena, fluctuation amplitudes and stationary distributions. The methods are illustrated on several gene network examples.

**Conclusion:**

Hybrid simplifications can be used for onion-like (multi-layered) approaches to multi-scale biochemical systems, in which various descriptions are used at various scales. Sets of discrete and continuous variables are treated with different methods and are coupled together in a physically justified approach.

## Background

At a molecular level, the functioning of cellular processes is unavoidably stochastic. Recent advances in real-time single cell imaging, micro-fluidic manipulation and synthetic biology have shown that gene expression and protein abundance in single cells are dynamic random variables submitted to significant variability [4-7].

Markov processes approaches to gene networks dynamics, originating from the pioneering ideas of Delbrück [8], capture diverse features of the experimentally observed variability, such as transcription bursts [4,6,7,9], various types of steady-state distributions of RNA and protein numbers [10,11], noise amplification or reduction [12,13]. In the case of molecular switches, random transitions between two or several metastable states with different transcriptional activities, limit the possibility of the corresponding genetic circuits to store cellular memory and generate variability [6].

Even the simplest stochastic model, such as the production module of a protein [11,14] contains tens of variables and biochemical reactions, and at least the same number of parameters. Direct simulation of such networks by Stochastic Simulation Algorithm (SSA) [15] is extremely time consuming. Network reverse engineering, model parameter identification, and design principles studies suffer from the same drawbacks when the full models are attacked with traditional tools. Various approximation methods were proposed, based on time discretization such as binomial, Poisson or Gaussian time leaping [16,17], or based on fast/slow partitions [18-21].

The time complexity of exact simulation algorithms scales with the number of jumps (reactions) per unit time. Currently available hybrid algorithms treat fast reactions as continuous variables, which significantly reduces the number of jumps [18-21]. Although computationally efficient, the use of slow reactions as discrete variables, and of rapid reactions as continuous variables, does not always provide a convenient description of the hybrid stochastic process. Indeed, the discrete/continuous structure of the state space of a hybrid molecular system is better expressed in terms of species components. Activity of some promoters and production of the corresponding proteins occurs in bursts [6,7]. In a bursting event, a steep increase of the number of transcripts is followed by relatively smoother exponential degradation. In this example, the natural candidates for continuous variables are the gene transcripts and products concentrations, while the states of the DNA promoters are the discrete variables. This picture also emphasizes the possibility for noise transfer from discrete, microscopic components, to continuous, mesoscopic, and closer to the phenotype, variables. In such processes, both microscopic and mesoscopic variables are noisy. At the mesoscopic scale, the stochastic fluctuations result from the intermittency of the dynamics and do not have a simple dependence on the numbers of molecules of the continuous components. Counter-intuitively, the noise amplitude could increase with the number of transcripts or proteins in a burst [6].

Stochastic intermittence or bursting occurs not only in transcription, but also in many other biochemical processes, such as action potential firing [22], blips in calcium signaling [23], possibly happloinsufficiency [24], etc.

Thus, a large class of molecular stochastic models can be approximated by stochastic hybrid processes in which some state components are discrete, while others are continuous. Processes of this kind have been intensively studied in relation to applications in technology (air traffic management, flexible manufacturing systems, fault tolerant control systems, storage models, etc.) [25-28]. Several classes of stochastic hybrid processes were studied, among which the most important are the switching diffusion processes and the piecewise deterministic processes. For switching diffusions, the continuous state component has continuous trajectories governed by stochastic differential equations, punctuated by jumps controlled by the discrete component. For piecewise deterministic processes, the continuous component follows deterministic ordinary differential equations between two jumps. The jumps can be changes of the parameters in the (stochastic) differential equations (switch of regime with no discontinuity in the trajectory), or instantaneous changes of the continuous variables (discontinuities of the trajectory), or both. Piecewise deterministic processes have been recently proposed as models for various biological systems [29,30]. Diffusion approximations (without jumps) of Markov processes justify approximate versions of the Gillespie algorithm, such as the *τ*-leap method [31]. A general approach allowing to obtain and classify various hybrid approximations that can be obtained from molecular Markov jump processes is still needed and represents a first result of this paper. Hybrid stochastic processes can be obtained by applying the law of large numbers (or the central limit theorem) to the continuous components. This will be performed here by using a partial Kramers-Moyal expansion of the chemical master equation. In many cases, this procedure provides only a moderate reduction of the number of stochastic jumps to be simulated. Indeed, remaining discrete components can jump rapidly, in which case the simulation by a hybrid process performs ineffectively.

The second result of this paper allows us to cope with frequent jumps of the discrete components by using averaging. Averaging techniques are widely used in physics and chemistry to simplify models by eliminating fast microscopic variables [32-34]. In our case, the microscopic variables are the discrete state components (for instance species present in low numbers) and the resulting approximations are averaged switched diffusion or averaged (piecewise) deterministic processes. Standard averaging techniques for discrete Markov chains [35] use state aggregation. However, state aggregation algorithms are effective only for Markov chains with a finite, small number of states. A more effective averaging method, that we present here, is cycle averaging in chemical reaction networks. Our method exploits the formal analogy between the quasi-stationarity assumption for fast deterministic linear cycles [36] and the stochastic averaging assumption for the same type of submodels.

Stochastic quasi-steady-state approximations presented in [37] combine singular perturbations for the master equations and Van Kampen's Ω-expansion [38] (first order Kramers-Moyal expansion) and are very close to our approach. However, we provide here a much more general setting and completely characterize the hybrid limits that result from this setting.

The stochastic dynamics of our simplified hybrid models provide good approximations of the un-reduced chemical master equation. We show rigorously elsewhere [39] that the simplified hybrid processes are weak approximations of the un-reduced Markov process. This means that all the statistical properties of the trajectories are approximated in distribution. In particular, steady state distributions of molecular species concentrations should be accurately reproduced. Also, distributions of intermittence times like the times between bursts in the production of mRNA or proteins, or the commutation time for stochastic switches are expected to be similar for the reduced model and for the initial model.

The structure of this paper is the following. In the Methods section we obtain hybrid simplifications for pure jump Markov processes and also propose a new averaging algorithm for these models. In the "Results and Discussion" section we present several examples of hybrid models obtained from stochastic chemical kinetics models, including a medium scale protein production model for which we demonstrate averaging.

## Methods

### Introductory notes on stochastic hybrid systems

Stochastic hybrid processes are couples of the type (*θ*(*t*), *x*(*t*)) where *θ*(*t*) is a discrete component (piecewise constant), *x*(*t*) is a vector with piecewise continuous components, and *t *is time. The discrete component *θ*(*t*) can be considered as a controlled Markov chain, whose transition matrix may depend on the continuous variable *x*(*t*). The discrete state space Θ (values of *θ*) can be finite or infinite. For instance, supposing that the discrete variables are *r *"rare" molecular species, whose numbers never go over a small value *N*, then Θ ⊂ {0,..., *N*}^*r *^has at most (*N *+ 1)^*r *^states. Different values of *θ *may also correspond to various states of a single molecule. For proteins with multiple phosphorylation sites the number of states is 2^*r *^where *r *is the number of sites. In the case of competitive transcriptional regulation, when *N *transcription factors are competing on *r *binding sites, the number of states is at most (*N *+ 1)^*r*^.

There are several possible ways to specify the transitions of the discrete variables. Generally, these can be given by the stochastic matrix *λ*_*i*, *j *_(*x*, *t*) where *λ*_*i*, *j *_*dt *+ *o*(*dt*) is the probability of a transition from the state *i *to the state *j*, between *t *and *t *+ *dt*. However, this representation is not handy when the matrices have very large dimension. In such cases, the usual molecular Markov jump process description is more appropriate. Then, possible transitions are specified by the stoichiometry vectors *γ*_*i*_. The intensity (propensity) function *λ*_*i *_(*θ*, *x*) represents the probability per unit time that a transition from *θ *to *θ *+ *γ*_*i *_takes place between *t *and *t *+ *dt *[26]. It follows that the probability for a transition (of any kind) between *t *and *t *+ *dt *is *λ*(*θ*, *x*) *dt *+ *o*(*dt*), where *λ *= ∑_*i *_*λ*_*i *_is the total intensity. The inverse of the total intensity represents the mean waiting time between two successive transitions. The transition *θ *→ *θ *+ *λ*_*i *_is chosen with probability *λ*_*i*_/*λ*.

Jumps can also occur in the continuous variables, in which case the size of the jump can be continuously distributed.

There are several possibilities for the evolution of the continuous variables leading to various classes of hybrid processes.

#### Piecewise deterministic processes

Piecewise deterministic processes were first formalized by [40] and found many applications in various areas ranging from industry to mathematical finance and biology.

The state of a piecewise deterministic process (PDP) is a couple *ζ *= (*θ*, *x*) where *θ *∈ Θ denotes the discrete variable and *x *∈ ℝ^*n *^denotes the continuous variable. A PDP is given by three local characteristics namely:

• a vector field *χ*_*θ *_(*x*) (flow function),

• a jump intensity *λ*(*θ*, *x*) and

• a transition measure *Q*^*T *^such that *Q*^*T*^(*θ'*, *x'*; *θ*, *x*) is the probability distribution of the post-jump positions (*θ'*, *x'*) ∈ Θ × ℝ^*n*^. Related to this is the jump distribution *Q*^*J *^giving the probability distribution of the jumps: *Q*^*J*^(*δθ*, *δx*; *θ*, *x*) = *Q*^*T*^(*θ *+ *δθ*, *x *+ *δx*; *θ*, *x*) (*Q*^*J *^is obtained from *Q*^*T *^by phase space translation).

Such a process involves deterministic motion punctuated by jumps. The lengths of the time intervals between successive jumps are random variables called waiting times *τ*_*i*_. On the time interval [*t*_*i*_, *t*_*i *+ 1 _= *t*_*i *_+ *τ*_*i*_] the discrete variable remains constant *θ *= *θ*_*i*_, while the continuous variable evolves according to the differential equation:

(1)

Let *ϕ*(*t*, (*x*_*i*_, *θ*_*i*_)) be the solution of the differential equation (1). We suppose that this solution exists and is unique for all *t *≥ *t*_*i*_. The waiting time is a random variable whose distribution reads [40]:

(2)

The reader can easily obtain Eq.(2). The probability not to jump in the time interval [*s*, *s *+ *ds*] is 1 - *λds *+ *o*(*ds*). Thus *P*[*τ*_*i *_> *s *+ *ds*] = *P*[*τ*_*i *_> *s*](1 - *λds*) + *o*(*ds*). By taking the logarithm we get log *P*[*τ*_*i *_> *s *+ *ds*] - log *P*[*τ*_*i *_> *s*] = -*λds *+ *o*(*ds*). Summing over *ds *leads to Eq.(2). The more familiar expression used in the Gillespie algorithm *P*[*τ*_*i *_> *t*] = exp[-*λ*(*θ*_*i*_) *t*] corresponds to the particular case when *λ *does not depend on the continuous variable.

After a deterministic evolution on the (*i *+ 1)-th interval, the discrete variable and the continuous variable can change according to:



where ,  are sampled from the jump distribution *Q*^*J*^.

The discrete variables are parameters of the differential equations describing the dynamics of the continuous variables. Notice that if  = 0 the only effect of a jump on the continuous variables is a change of regime (change of parameter in the differential equation). The trajectories of the continuous variables are globally continuous. However, the velocities of the continuous variables are discontinuous at jumps of the discrete variables. We call this possibility "switching". If  ≠ 0, then continuous variables can have instantaneous jumps and their trajectories are only piecewise continuous. We call this possibility "breakage". It is possible to have both "switching" and "breakage".

A PDP can be also defined by its generator [40,41], acting on functions *f *defined on the phase space:

(3)

The adjoint of the generator is used to obtain the Fokker-Planck equation [3,38,42] describing the time evolution of the probability distribution *p*(*θ*, *x*, *t*) of the process:

(4)

The structure of the generic PDP corresponds to the following simulation algorithm:

(1) Set the initial state condition *x*(*t*_0_) = *x*_0_, *θ *= *θ*_0_,

(2) Generate a random variable *u *uniformly distributed in [0, 1],

(3) Integrate the system of differential equations



between *t*_*i *_and *t*_*i *_+ *τ*_*i *_with the stopping condition *F *(*t*_*i *_+ *τ*_*i*_, *θ*_*i*_, *x*_*i*_) = *u*,

(4) Generate a second uniform variable *v *and use it to randomly choose the jumps (, ) (the decision is made in the same way as in the Gillespie algorithm [15]),

(5) Change the system state (*θ*_*i*_, *x*(*t*_*i*_)) into (*θ*_*i *_+ , *x*(*t*_*i*_) + ),

(6) Reiterate the system from 2) with the new state until a time *t*_max _previously defined is reached.

#### Hybrid diffusions

A rather general class of hybrid diffusions has been proposed in [26]. However, in that setting, jumps of continuous variables are commanded by the hitting of some predefined phase space sets, which is not our case. We propose a simpler and different setting, which is natural for biochemical systems. In our setting, a hybrid diffusion is defined by:

• a vector field *χ*(*θ*, *x*) (drift or flow function),

• a diffusion matrix *σ*(*θ*, *x*),

• a jump intensity and a transition measure defined like for PDPs.

Between two consecutive jumps at *t*_*i *_and *t*_*i *_+ *τ*_*i*_, the continuous variables *x*(*t*) satisfy the Itô stochastic differential equations [43]:



where *W*_*j *_(*t*) are independent one-dimensional Wiener processes.

The waiting time *τ *is determined like for PDPs. One should integrate the system:



with the initial condition *F*(*t*_*i*_) = 1, *x*(*t*_*i*_) = *x*_*i *_and the stopping condition *F*(*t*_*i *_+ *τ*_*i*_) = *u*, where *u *is a random variable, independent from (*θ*_*i*_, *x*_*i*_), uniformly distributed on [0, 1].

The generator for such a process is:

(5)

where "." stands for the scalar product and ":" stands for the double contracted tensor product, i.e. .

Notice that hybrid diffusions contain PDPs as the particular case when diffusion is zero (*σ *= 0).

### Hybrid simplification of Markov pure jump processes (MPJP)

#### Markovian stochastic chemical systems

Most of the existing numerical methods for stochastic chemical systems are based on the representation of the chemical reaction system as a Markov pure jump process (MPJP), that corresponds to Gillespie's [15,44] stochastic simulation algorithm (SSA). The state of a system of *n *chemical species *A*_1_,..., *A*_*n *_is a vector *X*(*t*) whose components are the numbers of molecules from each species. The state space of the process is *E *= ℤ^*n*^. From now on, we shall use upper case letters *X *for numbers of molecules, and lower case letters *x *= *X*/ for concentrations, where  is the reaction volume, typically the volume of the cell's compartment.

For each reaction,

(6)

we have the jump *γ*_*i *_= *β*_*i *_- *α*_*i *_∈ ℤ^*n*^, *i *= 1,..., *n*_*r *_(*n*_*r *_is the number of reactions). We consider equally the reverse reaction, corresponding to the jump -*γ*_*i*_. The intensities and the transition measures for the Markov jump process are:

(7)

(8)

*V*_*i*_, *V*_-*i *_are the reaction rates and probabilities  satisfy . Here *δ*_*X *_is the Dirac measure centered on *X*, and *X' *is the post-jump position.

Several choices are possible for the reaction rates [45]. The most popular is the mass action law, when rates are polynomial functions of concentrations:

(9)

As for any homogeneous Markov process, we can characterize this process by its generator [41], whose action on test functions *f *defined on the state space *E *is:

(10)

The adjoint of the generator defines the chemical master equation which describes the time evolution of the probability distribution *p*(*X*, *t*) in phase space.

(11)

Notice that hybrid diffusions or PDPs with no continuous components are Markov pure jump processes.

#### Partial Kramers-Moyal expansion

Diffusion approximations (Langevin dynamics) for stochastic chemical kinetics was proposed by [31] and was rigorously justified by [46,47]. This approximation is valid when the numbers of molecules of all species are much larger than one. It concerns for instance the thermodynamic limit  → ∞ where  is the volume of a well stirred reactor. Then, the diffusion approximation applies to intermediate scales  in phase space. Formally, this approximation can be obtained by the Kramers-Moyal expansion [1-3], which is the second order Taylor expansion of the master equation with respect to jumps divided by the volume; in the first order one gets the deterministic dynamics, and in the second order the Langevin dynamics. In [29], we have used partial Kramers-Moyal expansions to obtain hybrid approximations of MPJP. The possibilities of this method are examined here in more generality.

First, let us partition the species of the system into two sets *X *= (*X*_*D*_, *X*_*C*_), where *X*_*D *_represents the species in small numbers and *X*_*C *_the species in large numbers. Note that this partition is different from reaction based partitions used in other works [18,20,21,48-50]. All these works have as objective the reduction of simulation time. With respect to this objective, many schemes that avoid individual simulation of rapid reactions by regrouping them and by treating the corresponding channels as continuous variables in "fluid" limits, are more or less equivalent. We fix ourself also another objective, which is to find a natural representation of the simplified processes. A species based partition is more suitable for this end. Let *M *be the set of reactions in which every reversible reaction contributes with two reactions, one for each direction. The species partition determines a partition of *M *into three sets . The reaction partition distinguishes between the reactions whose reactants and products are in *X*_*C *_(), reactions whose reactants and products are in *X*_*D *_() and reactions with at least a reactant or a product in *X*_*D *_and at least a reactant or product in *X*_*C *_(). We should emphasize that although  reactions are rapid, some other rapid reactions can be contained in  or .

The second step towards the hybrid processes is to rescale the continuous variables by dividing them with the volume and obtaining *x*_*C *_= *X*_*C*_/. The *X*_*D *_variables will not be rescaled and will be considered discrete. Their evolution is given by discrete transitions.

The third step is to consider a second order Taylor expansion of the master equation, for the continuous variables *x*_*c *_and with respect to the small parameters *γ*_*i*_/. This expansion can be performed equivalently on the generator (10) or on the master equation (11). For simplicity, we use the generator. The test functions depend now on two variables (*x*_*C*_, *X*_*D*_) and are considered to be twice differentiable with respect to the continuous variables *x*_*C*_.

Let us consider that rates of reactions in  are proportional to the volume (this follows from mass action law), , *i *∈ . The second order Taylor expansion of (10) with respect to *x*_*C *_reads:

(12)

where ⊗ is for tensor product ((*γ *⊗ *γ*)_*kl *_= *γ*_*k *_*γ*_*l*_).

The approximated generator corresponds to hybrid diffusions with drift function  and diffusion matrix . As it can be easily seen, the drift and diffusion coefficients for the variables *x*_*C *_do not depend on the discrete variables *X*_*D*_. Thus, switching is not present in this approximation and discrete variables can be just forgotten if not observed. Furthermore, jumps  vanish in the limit  → ∞, implying that continuous variables have no instantaneous jumps (no breakage). However, switching and breakage is present in many examples in molecular biology. How is this possible?

In order to cope with the possibility of switching and breakage we need to consider that some reactions from  exhibit particular properties. We can consider two types of special reactions:

1. reactions in  that are frequent enough to change the continuous variables and induce switching,

2. reactions in  with a large stoichiometric vector, that induce breakage.

These reactions will be referred to as "super-reactions". The first type of super reactions are denoted by the set  and the second type of super-reactions are denoted . As a consequence, we distinguish two types of hybrid approximations following which one of the two "super-reactions" are present in the mechanism.

### A. Hybrid approximations with switching

This first case of approximate process is induced by "super-reactions"  with stoichiometric vectors of order one, but with rates that scale with the volume: , *i *∈ . Reactions from  induce rapid transitions of the continuous variables. The set  is considered to be empty. For this approximation, even if the rates of the "super-reactions" depend on the discrete variables, we suppose that the "super-reactions" do not change the discrete variables. In other words

(13)

If this condition fails, another type of approximation arises: some discrete variables change rapidly. The resulting process will be an averaged hybrid process.

The evolution of the discrete variables is given by slow jumps (reactions from ). The component *x*_*C *_has rapid jumps but their amplitude is relatively small. Thus, between two consecutive transitions of the discrete variables, the continuous variables can be approximated by a smooth deterministic trajectory. The continuous variables have continuous trajectory, however, discontinuous velocities. The first order of the Kramers-Moyal expansion corresponds to the *piecewise deterministic approximation *defined by the flow function

(14)

the jump intensity

(15)

and the jump probability

(16)

where ,  are the projections of *γ*_*i *_on continuous and on discrete components, respectively. The relation (15) clearly shows that the jump intensity depends both on continuous and on discrete variables. Nevertheless, only the discrete variables are changed by jumps.

The second order of the Kramers-Moyal expansion corresponds to the *hybrid diffusion approximation *whereas the diffusion matrix reads:

(17)

### B. Hybrid approximations with breakage

This type of approximation is induced by the "super-reactions" with a large stoichiometric vector (i.e. reactions ). We suppose also, that the set  is empty. In other words, to have this approximation, we suppose that at least one reaction *i *in the set  has a large stoichiometric vector (*λ*_*i*_)_*j*_/ is comparable with |*x*_*j*_| for *i *∈  and for some species *j *∈ *C*. A reaction  can change both continuous and discrete components. The main difference consists in the fact that a reaction  significantly change, in one step, the concentration of the *x*_*C *_component.

The reactions of the type  do not contribute to the deterministic flow. They only appear as jumps. As before, we write down the main characteristics of the approximated PDP process (first order Kramers-Moyal expansion), namely the flow function

(18)

the jump intensity

(19)

the jump probabilities for the discrete variables

(20)

and the jump probability for the continuous variables

(21)

If the super-reactions *i *∈  have  ≠ 0, jumps occur simultaneously in the continuous and in the discrete variables.

The deterministic flow is defined by the flow function *χ*(*x*_*C*_) (Eq.(18)) which does not depend on the discrete variable *X*_*D*_. In this case there is no switching. In the second order of the Kramers-Moyal expansion, we include phase space diffusion, which contains contributions only from reactions of :

(22)

If both sets ,  are not empty, then both types of discontinuities can appear in the trajectory of *x*_*C*_:

• switching: discontinuity of velocity (jump of *X*_*D*_), with no discontinuity of trajectory,

• breakage: discontinuity of trajectory (jump in some variable *x*_*C*_).

The first discontinuities are induced by reactions from , while the latter are induced by reactions from .

### C. Hybrid approximations with singular switching

Although mathematically distinct, breakage and switching could be physically indistinguishable in certain cases. Indeed, the jump of the continuous variable can result from the repeated application of one or several very fast reactions from . We can obtain processes with breakage as singular limits of processes with switching when very short lasting steep variations alternate with long lasting slower variations of the continuous variables.

Let us consider that there is a subset of extremely rapid super-reactions  such that:



where 0 <*ϵ *<< 1 is a small positive parameter, and where  are discrete species, substrates of the reaction *i *∈ .

Like for reactions , we consider that  = 0, for all *i *∈ .

For each reaction *i *∈  we consider that there are two subsets of reactions in :  contains reactions that produce the species  and  contains reactions that consume the species . We define . We consider that all reactions in  are rapid:



First, we apply the Kramers-Moyal expansion for large  and obtain a switched PDP. The flow function of this PDP reads:



where



The jump intensity of the PDP reads



We show in the Additional File 1 that, in the limit *ϵ *→ 0, the switched PDP converges to a PDP having the following generator:

(23)

where  is a probability density satisfying  and Φ(*s*; *x*, *X*_*D*_) is the solution of the differential equation .

We recognize above the generator of a PDP with breakage. Contrary to the previous case of breakage resulting from super-reactions , the breakage size is now continuously distributed. The switch transitions  disappear in the singular limit (the substrates of the reactions  remain practically all the time in the state  = 0).

#### Practical criteria for identifying small parameters and super-reactions leading to piecewise deterministic approximations

The law of large numbers is applicable in the limit  → ∞. Certainly, in cell biology, the idea of infinite volumes should be considered with care. For this reason we will replace this condition by a set of easy to check criteria concerning relative orders of parameters of the models. These criteria will concern piecewise deterministic approximations. Criteria for hybrid diffusion approximation, involving central limit theorem, will not be discussed in this paper.

Our criteria are relative to two reference quantities. The first reference is a large number *N *representing a lower bound for the numbers of molecules of continuous species. The second reference is a time *τ*, which is a lower bound both for the characteristic times of the deterministic dynamics of the continuous variables *τ*_*C *_and for the average time between two successive jumps of the discrete variables *τ*_*D*_, namely *τ *= min(*τ*_*C*_, *τ*_*D*_). *τ *can be related to kinetic parameters by methods exposed in [51,52].

The applicability of the law of large numbers to continuous variables implies two conditions. The first condition is that jump sizes should be small with respect to the numbers of molecules, i.e.  <<*N *for all *i *∈ . The second condition is that the number of jumps of the continuous variables on the timescale *τ *should be big, i.e. *V*_*i *_*τ *>> 1 for all *i *∈ . The two conditions are fulfilled simultaneously in the limit  → ∞ because *N*, *V*_*i *_scale with  and , *τ *do not depend on .

A similar condition must be satisfied by super-reactions of the type 1: *V*_*i *_>> *τ*^-1 ^also for all *i *∈ . The super-reactions of the type 2 do not satisfy the small jump condition, i.e.  must be comparable to *N *(not much smaller) for all *i *∈ .

In order to have singular switching we need a new small parameter *ϵ *<< 1 and the following set of conditions:

**a) **There are super-reactions of the type 3. These are just very quick super-reactions of the type 1.

**b) **Super-reactions of the type 3 act only on a laps of time *τ*_*ϵ *_= *ϵτ *i.e. *V*_*i *_= (*ϵτ*)^-1 ^>> *τ*^-1 ^for *i *∈ . Reactions in  that inactivate super-reactions of type 3 could thus be as frequent as reactions in  and in  (but not as quick as reactions in ).

**c) **Super-reactions of the type 3 are quick enough to produce breakages comparable to *N *during the time *τ*_*ϵ*_, i.e. *V*_*i *_>> (*ϵτ*)^-1 ^for *i *∈ .

The conditions of the type *V*_*i *_>> *τ*^-1 ^can be simplified if the reactions are first order with respect to discrete species. In this case *V*_*i *_= *k*_*i *_*X*_*D *_with *X*_*D *_≈ 1, therefore the condition is *k*_*i *_>> *τ*^-1^. All these criteria are summarized in the Table 1.

**Table 1:** Practical criteria to be satisfied by various reactions.

**Reaction set**	**Approximation**	**Condition**
	hybrid all	<<*N *and *V*_*i *_>> (*τ*)^-1^
	hybrid with switching	<<*N*, *V*_*i *_>> *τ*^-1^
	hybrid with breaking	~ *N*
	hybrid with breaking as singular limit	*V*_*i *_= (*ϵτ*)^-1 ^>> *τ*^-1^
	hybrid with breaking as singular limit	*V*_*i *_>> (*ϵτ*)^-1^
cycling reactions	averaged hybrid	*k*_*lim *_>> (*τ*_*C*_)^-1^
branching reactions	averaged hybrid	*k *<<*k*_*j*_, *j *cycling

See text for definitions of various reaction sets. *τ*_*C*_, *N *are typical timescale and number of molecules of the continuous components, respectively. *τ *= min(*τ*_*C*_, *τ*_*D*_), where *τ*_*D *_is the mean time between two successive jumps of the discrete components. *V*_*i *_are the average numbers of reactions per unit time (intensities of jump Markov processes), *k *are first order kinetic constants and *γ*_*i *_are the stoichiometric vectors.

### Averaging for stochastic chemical kinetics

The performance of the hybrid algorithm can be very bad when the discrete mechanism contains rapid cycles which effectuate many reactions on the deterministic timescale *τ*_*C *_(this is the timescale on which the continuous variables have significant variation). Indeed, in this situation the deterministic solver is artificially sampling the interval between two discrete cycle reactions. First, this leads to unreasonable increase of the simulation time. Second, the condition on the number of jumps of continuous variables is not satisfied and the hybrid approximation is not accurate. In this case, the hybrid scheme performs worse than SSA. It is therefore important to eliminate rapid cycling from the system before implementing numerical schemes for the hybrid approximations. This can be done by averaging.

Averaging principles are widely used for deterministic (see classical textbooks [53,54] more recently revisited in the non-autonomous case by [55]) and stochastic (stochastic differential equations [56], Markov chains [35,57]) systems.

The classical averaging idea is to identify fast ergodic (that, starting in any value, can reach in finite, small time any other value in a given phase space set) variables and to average the dynamics of the slow variables with respect to the quasi-stationary distribution of the fast variables. An important difficulty is to identify the right slow and fast variables. For perturbed Hamiltonian dynamical systems the action-angle variables provide the natural framework for averaging [53].

In the case of chemical kinetics, there are two kinds of slow variables that should be taken into account for averaging. The first kind are just slow components (discrete components that change infrequently or continuous components with large deterministic timescale). The second kind are linear combinations of components that are conserved by the fast cycling dynamics. These new slow variables, that play a role similar to the action variables in Hamiltonian systems, provide new "aggregated" or "lumped" species in the averaged system. Notice that aggregation of states has been used for averaging Markov chains [35,57]. The aggregation of species that we propose here adapts this type of argument to the case of chemical kinetics. Variable aggregation for simple deterministic reactive models has been used in relation to applications in ecology (see for instance [58]). In [36,52] we proposed a general solution for simplification of reaction networks, that works for any linear mechanism with well separated constants. In this algorithm, species aggregation is systematically applied to hierarchies of rapid cycles. We show here that the same algorithm can be effectively used also in the stochastic case.

#### Averaging principles for reaction mechanisms

Averaging can be applied to multi-scale reaction mechanisms. This procedure leads to averaged hybrid models. In order to apply averaging, one should first identify a sub-model of discrete components, satisfying the following conditions:

**C1) **the dynamics of the sub-model is much faster than the dynamics of continuous and of other discrete, slower modes.

**C2) **the dynamics of the sub-model is ergodic: starting with any state, the system can reach any other state in finite time.

The general procedure to obtain averaged hybrid simplifications is described in the Additional File 1. Some cases are particularly interesting.

The first case corresponds to *fast discrete cycles producing continuous species*. In this case rapid super-reactions  change both discrete and continuous components (Eq.(13) is not valid). The discrete components that are affected by such reactions are fast discrete variables. In the resulting hybrid model, both the continuous dynamics and the slow discrete reaction rates should be averaged with respect to the fast discrete variables.

The second case corresponds to *fast, purely discrete cycles*. In this case, some of the cycles of the sub-mechanism  are at least as fast or faster than reactions . The other reactions of  are much slower. In the resulting model, rates of the slow reactions of  should be averaged with respect to the fast discrete variables.

In both cases, one needs the steady probability distribution for the fast discrete sub-model. All the slow processes will be averaged with respect to this distribution. The calculation of the steady probability distribution can be easily done only when the sub-model is linear. Considering linear sub-models has also another advantage. In this case, averaging and aggregation lead to a coarse-grained reaction mechanisms. For these reasons, we have developed an algorithm for linear sub-models. Of course, gene network models are generally nonlinear. However, this does not mean that all the parts of such models behave nonlinearly. Many sub-models, in particular monomolecular cycles, can be simplified by averaging methods that we designed for linear networks.

#### Cycle averaging for linear sub-models

##### Linear sub-models

The are two types of linear reaction networks: monomolecular networks and first order networks. The structure of monomolecular reaction networks can be completely defined by a simple digraph, in which vertices correspond to chemical species *A*_*i*_, edges correspond to reactions *A*_*i *_→ *A*_*j *_with rate constants *k*_*ji *_> 0. For each vertex, *A*_*i*_, a positive real variable *c*_*i *_(concentration) is defined.

The deterministic kinetic equation is

(24)

First order reaction networks include monomolecular networks as a particular case, and are characterized by a single substrate and by reaction rates that are proportional to the concentration of the substrate. First order reaction networks can contain reactions that are not monomolecular, such as *A *→ *A *+ *B*, or *A *→ *B *+ *C*. We shall restrict ourselves to pseudo-conservative first order reactions, ie reactions that do not change the total number of molecules in a given submechanism (*A *→ *A *+ *B *reactions are allowed, provided that *B *is external to the submechanism; similarly *A *→ *B *+ *C *reactions are allowed, provided that either *B *or *C *is external to the submechanism). With such constraints, the total number of molecules in the sub-mechanism is conserved and the kinetic equations are the same as (24). Degradation reactions can be studied in this framework by considering a special component (sink), that collects degraded molecules. Further release of the constraints is possible. For instance, the system can be opened by allowing constant (or slowly variable) production terms in Eq.(24). These terms will change the steady state, but will not influence the relaxation times of the system. The linear sub-mechanisms can be considered as part of a nonlinear network, given fixed (or slowly changing) values of external inputs (boundaries). For instance, even in systems of binary reactions, one can define pseudo-monomolecular reactions when one of the substrates of the binary reaction is not changing (or changing slowly). This condition can be fulfilled if the substrate is in excess, for instance.

The stochastic dynamics of a unique molecule in such linear reaction network is given by the probability *p*(*j*, *t*) that the molecule is in *A*_*j *_at the time *t*. We can easily show that the master equation for *p*(*j*, *t*) is the same as the deterministic kinetic equation (24). Considering only one molecule does not restrict generality because when several molecules are present in a linear network, these behave independently. Thus, the simplification algorithm proposed for deterministic networks [36,52] can be also applied to stochastic networks [59]. The algorithm is based on a set of operations transforming the reaction graph into an acyclic digraph without branching (no graph component is the substrate of more than one reaction). For such type of graphs the eigenvectors and eigenvalues of the kinetic matrix can be straightforwardly calculated, which completely solves the problem of deterministic dynamics. We could follow precisely the same approach to simplify and then solve the stochastic dynamics. However, we argue here that applying only a few steps of the algorithm is enough for effectively reducing computational time of the SSA method.

##### Cycle averaging algorithm

Let us recall that the reduction method presented in [36,52] uses two types of operations acting on the reaction graph.

I. Construction of an auxiliary network (dominance). For each node *A*_*i *_of a linear sub-mechanism, let us define *κ*_*i *_as the maximal kinetic constant for reactions *A*_*i *_→ *A*_*j*_: *k*_*i *_= max_*j*_{*k*_*ji*_}. For the corresponding *j *we use the notation *ϕ*(*i*): *ϕ*(*i*) = arg max_*j*_{*k*_*ji*_}. An auxiliary reaction network  is the set of reactions *A*_*i *_→ *A*_*ϕ*(*i*) _with kinetic constants *κ*_*i*_. In such a network there is no branching: if several reactions consume the same component *A*_*i*_, only the quickest one is kept and all the other discarded.

II. Glueing cycles (aggregation). Rapid cycles are replaced by a single node. Constants of reactions leaving these cycles are renormalized according to an averaging principle (see the Additional File 1).

In order to present the simplification algorithm let us use two simple examples.

First, let us consider a chain of molecular reactions *A*_1 _→ *A*_2 _→ ... *A*_*m*_. The reaction rate constant for *A*_*i *_→ *A*_*i*+1 _is *k*_*i*_. All rate constants are considered well separated, i.e. either *k*_*i *_<<*k*_*j *_or *k*_*i *_>> *k*_*j *_for any *i *≠ *j*.

The smallest rate constant in the chain is called limiting, and denoted by *k*_lim_. If 1/*k*_lim _<<*τ*_*C *_(rapid chain), then all molecules *A*_1 _are transformed into molecules *A*_*m *_on a timescale much quicker than the deterministic time *τ*_*C*_. We can thus ignore the chain reactions and consider that the entire mass of the chain is practically always in *A*_*m*_. This is equivalent to considering the chain at quasi-stationarity because the steady state probability distribution of a chain is a Dirac delta measure localized at the end of the chain. However, if we do not simplify chains, then simulating them by Gillespie's SSA will not be computationally expensive because the mass of the chain is transferred to the end of the chain *A*_*m*_in a number of steps that is relatively small (this is bounded by the total mass of discrete species multiplied by the longest chain length).

As a second example, let us consider the cycle *C *be the following sequence of mono-molecular reactions *A*_1 _→ *A*_2 _→ ... *A*_*m *_→ *A*_1_. Let all rate constants be well separated and the smallest one be *k*_*lim *_like before.

We add to the cycle one branching reaction; this transforms *A*_*j *_a component of the cycle into *B *a component exterior to the cycle.

We consider the following distinct situations:

**(I) **the branching reaction is *A*_*j *_→ *B *of rate constant *k *and *k *>> *k*_*j*_,

**(II) **the branching reaction is *A*_*j *_→ *B *and *k *<<*k*_*j*_,

**(III) **the branching reaction is *A*_*j *_→ *A*_*j *_+ *B*, or

**(IV) **the branching reaction is *A*_*j *_→ *A*_*j*+1 _+ *B *of rate constant *k*_*j*_.

In the situation (I) the exit reaction is faster and dominates the cycling reaction *A*_*j *_→ *A*_*j*+1_. According to the rule for auxiliary networks in this case (that we call "broken" cycle) the cycle can be opened (by eliminating the cycling reaction *A*_*j *_→ *A*_*j*+1_) and the resulting multiscale dynamics is the one of a chain; we recover the previous example and in this case we can safely decide to do nothing.

In the situation (II) the exit reaction is much slower than the cycling reaction. In this case the molecules inside the cycle have rapid transformations and the mass distribution inside the cycle can be considered to reach quasi-stationary distribution. We call this cycle "unbroken".

As discussed in [36,51,52], the relaxation time of a cycle with separated constants is the inverse of the second slowest rate constant *k*^(2) ^>> *k*^(1) ^= *k*_*lim*_. To understand this, one should consider the two possible paths to equilibrate a cycle, one passing by the slowest step and the quicker one passing by the second slowest step: the quicker short-cuts the first one. Thus, a cycle can be considered quasi-stationary if *k*^(2) ^>> 1/*τ*_*C*_. A non-averaged fast cycle is computationally expensive in SSA, if a molecule can perform a huge number of steps along the cycle on the timescale *τ*_*C*_. The corresponding condition involves the quasi-stationary flux (not the relaxation time) and reads *k*^(1) ^= *k*_*lim *_>> 1/*τ*_*C*_.

From a quasi-stationary cycle, the mass is lost stochastically, but slowly by the branching reaction. The intensity of the loss process can be calculated by replacing *X*_*j *_by its average with respect to the quasi-stationary distribution of the cycle. The average of *X*_*j *_is  = *N*(*t*) *k*_lim_/*k*_*j*_, where *N*(*t*) is the total mass inside the cycle . We obtain the average intensity  = *N*(*t*) *kk*_lim_/*k*_*j*_.

In the situations (III) or (IV) the average intensities of the branching reactions are  = *N*(*t*) *kk*_lim_/*k*_*j *_and  = *N*(*t*) *k*_lim_, respectively.

All these operations can be presented as a:

#### Simplification algorithm

Input:

a first order reaction mechanism G with separated kinetic constants.

Output:

a simplified first order reaction mechanism.

**while **there are fast unbroken cycles

**for each **cycle **in **G *not containing reactions of the type (I) having a sufficiently intense flux *(*k*_*lim *_>> 1/*τ*_*C*_) **do**

**1**: "glue" the cycle into a single node *C *having the total mass *N*;

**2**: replace the exit reaction of the type (II) *A*_*j *_→ *B *of rate constant *k *by a reaction *C *→ *B *of effective constant *k' *= *kk*_lim_/*k*_*j*_;

**3**: replace the reaction of the type (III) *A*_*j *_→ *A*_*j *_+ *B *or rate constant *k *by a reaction *C *→ *C *+ *B *of effective constant *k' *= *kk*_lim_/*k*_*j*_;

**4**: replace the reaction of the type (IV) *A*_*j *_→ *A*_*j*+1 _+ *B *of rate constant *k*_*j *_by a reaction *C *→ *C *+ *B *of effective constant *k' *= *k*_lim_;

Imposing the "glueing" condition on the cycling flux and not on the cycle relaxation time allows to iterate the cycle averaging algorithm until no more fast unbroken cycles remain. This would not be possible when adopting a relaxation time criterion to perform averaging. Indeed, the relaxation time of a cycle is given by the second slowest constant. This leaves place for a counter-intuitive possibility: one can have slow cycles embedded into rapid cycles. For instance, a fast cycle whose nodes are "glued" cycles can have a slower node as the beginning of its limiting step. Indeed, the internal constants of this node are necessarily more rapid than the limiting step of the large cycle, but can be slower than the second constant which gives the relaxation time of the large cycle. The slow scales are lost by glueing. In order to recover the full multi-scale dynamics, a restoration operation has been considered at the end of the algorithm presented in [36,52]. In this operation, all "glued" cycles are restored without their limiting steps. Thus, slower cycle sub-dynamics can be recovered. Restoration is not needed for glued cycles satisfying the flux condition *k*_*lim *_>> , because these are automatically faster than the timescale *τ*_*C*_. Our averaging method works for mechanisms with well separated constants. Generalization for partially separated constants are possible. For instance, one can consider cycles containing reactions with equivalent constants, but such that for each node of the cycle, the constant of the branching reaction is much smaller than the constant of the cycling reaction.

## Noisy networks and design rules for hierarchies of cycles

As we have shown in the previous sections, intrinsic noise in biochemical networks is generated at a "microscopic level" by the discrete variables and can be observed at the "mesoscopic level" of the continuous variables either as switching, or as breaking events. Thus, when there are no discrete variables (all species are in large numbers), there is no intrinsic noise. Also, if the switching events are much faster than the deterministic time scale, averaging principles apply and noise is not transmitted to the continuous variables: the deterministic approximation is again recovered. The only way to transmit noise by switching to the mesoscopic level is by intermittency and this needs particular combinations of slow reactions that change the values of the discrete species and frequent reactions that change the continuous species. Intrinsic noise transmission from micro- to meso-scopic level results from certain (not all) combinations of low numbers, fast and rapid timescales. Some general design rules relating topology to dynamic properties relative to noise can be derived from our approach.

By hierarchies of cycles we understand reaction mechanisms containing cycles connected by branching reactions forming higher level cycles. The nodes of higher level cycles are "glued" cycles from the lower levels. Hierarchies of cycles in discrete variables have interesting properties with respect to noise production. Generally, in cycle hierarchies, effective constants of branching reactions are at least as slow or slower than the limiting step (slowest reaction) of the node (glued cycle) from which they start. Coupling cycles into hierarchies is a way to produce slower and slower reactions from initially rapid reactions and generate thus intermittency.

As a possible design rule, we could state: exit reactions of the type (II) or (III) (but not (IV)) generate intermittency when the exit node is not the beginning of the limiting step in some unbroken fast cycle.

Indeed, unless *k*_*j *_is the limiting step in the cycle, one has *k*_lim_/*k*_*j *_<< 1. Then, the average intensity of the exit reaction of the type (II) or (III) is weak and could represent a source of intermittency in the system. This situation should be avoided for less noise in the system, or created when noise is wanted.

An example of how this rule applies to the biochemistry of bacteria will be given below. A systematic investigation of the possibilities of this class of design rules will be presented elsewhere in relation to synthetic biology.

## Results and Discussion

### Methodology to obtain hybrid simplifications

We demonstrate how hybrid simplifications can be obtained from Markov pure jump models for gene networks. The simplified models preserve the main stochastic properties of the initial complex models and can replace these models in simulations.

The simplification procedure consists of four successive steps:

**I **Identification of discrete and continuous variables, partition of the reactions.

**II **Cycle averaging.

**III **Identification of super-reactions.

**IV **Construction of the hybrid simplifications.

In order to justify the utility of our approach we show by examples that all types of hybrid processes that we have discussed are represented in gene networks models.

To introduce the examples we employ the following notation. Reactions are numbered by integers. If the *i*-th reaction is reversible, then  is the *i*-th reaction in the forward direction and  the *i*-th reaction in the reverse direction. Irreversible reactions are denoted simply *R*_*i*_.

The rate constants units are *s*^-1 ^for monomolecular and *s*^-1^(M)^-1 ^for binary reactions. In order to obtain pseudo-monomolecular rate constants from binary reaction constants with slowly varying substrates *X *we have used the following formula:



where *N*_*A *_is the Avogadro number and  is the cell volume. For a bacterium, *N*_*A *_ ≈ 1(n M)^-1^.

Similarly, the reaction rates are calculated as



We discuss two simple hybrid models, one with switching the second one with breakage, then two more complex models that need averaging. All our simulations were performed using MATLAB 2008 in a Windows XP32 environment with a dual core INTEL 6700, 2.65 GHz processor.

### Hybrid model with switching: Cook's model

The simplest model with switching has been introduced by Cook [24] as a model for haploinsufficiency phenomena. This model can be described by the following system of biochemical reactions:



In this model, *G*, G* represent inactive, respectively active states of the gene. In the active state, the gene produces some protein *P*. Let *G*_0 _be the gene copy number, which is a conserved quantity of the dynamics *G *+ G* = *G*_0_. The haploinsufficiency regime corresponds to a small value of *G*_0_. For the simplicity of the argument we consider *G*_0 _= 1.

Let *X *= (*X*_*D*_, *X*_*C*_) be the state vector. We notice that, the partitions of the species is the following: the species in large number *X*_*C *_= {*P*}, and the species in small number *X*_*D *_= {*G*, G*}. This partition of the species defines a partition of reactions. With the above notations we get:



The deterministic timescale is *τ *= (*k*_3_)^-1^. The stoichiometric vectors have all lengths of order one, much smaller than *N *(the number of proteins).

For the parameters values used in [24] we have *k*_2_/*k*_3 _>> 1 and *R*_2 _is a super-reaction of the type . The reaction *R*_3 _satisfies *V*_*i *_= *N k*_3 _>> *k*_3 _= *τ*^-1^, which means that the law of large numbers can be applied to the continuous variable.

In the first order of the Kramers-Moyal development we obtain a PDP approximation with switching. In the following, we will denote by *x *= [*P*] the continuous variable and by *θ *= G* the discrete variable. This model is a piecewise deterministic process with state space (*θ*, *x*) ∈ {0, 1} × ℝ.

The flow function *χ*(*θ*, *x*) is given by



and the jump intensity *λ *is defined by



**Figure 1:**
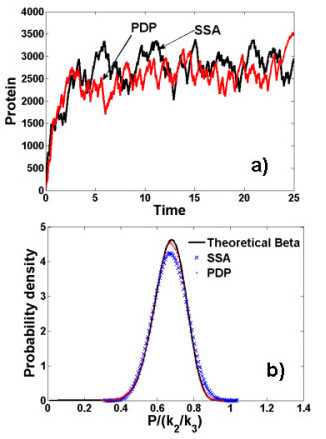
**Cook's haploinsufficiency model, an example of PDP with switching**. a) Time evolution of the protein concentration using Gillespie SSA method and PDP approximation. b) Estimated Gillespie steady probability distribution vs. estimated steady piecewise-deterministic (PDP) probability distribution. The theoretical beta distribution for the PDP is added.

The resulting PDP is the same as ON-OFF systems studied in operational research [25]. The model has been proposed as abstract model for stochastic protein production in several other places [29,60]. With the PDP description of the system and using the hybrid algorithm introduced in the section Methods, we draw a possible time evolution of this system (see Figure 1a). The same initial condition was used when the Gillespie algorithm was implemented for the model described above. The simulation time to generate a trajectory using a PDP approximation, was 2.6 seconds, while with the Gillespie algorithm the simulation time was 14.5 seconds in the average. The trajectories look qualitatively the same: production intervals are followed by degradation intervals, with no discontinuity in the continuous variable.

In order to quantitatively test the accuracy of the PDP algorithm, we have computed the stationary distribution of the number of proteins using a piecewise deterministic simulation and compared it to the estimated distribution from Gillespie trajectories.

The numerical methods to estimate stationary distributions are described in the Additional File 1. The theoretical curve for the PDP model is a beta distribution. The variable *x *= *P*/(*k*_2_/*k*_3_) follows the Beta distribution , *B *is the Beta function [29]. The result of various comparisons is represented in Figure 1b.

Cook's model operates in the region *k*_2 _>> *k*_*m*1_, which corresponds to a broken cycle in the discrete variables. If the opposite inequality is valid *k*_2 _<<*k*_*m*1_, then cycle averaging is needed. The cycle  should be glued to a point  of mass  = *G*_0 _= 1 and the branching reaction *R*_2 _becomes , where . The resulting model is a birth and death model with effective birth rate  and death rate constant *k*_3_. Provided that *k*_2_/*k*_3 _>> 1 (meaning that *R*_2 _remains a super-reaction of the type ), the model can be approximated by a completely deterministic process with flow given by the averaged flow function *χ*(*x*) = -*k*_3 _*x *+ .

### Hybrid models with breakage: neuroscience and bacterium operator sites

Neural systems exhibit stochastic behavior. Stein [61,62] proposed a simple Markovian model for the evolution of a neuron membrane potential. In this model, discontinuous random jumps of the potential are followed by exponential decay. We do no discuss here how to obtain this hybrid model from a microscopic pure jump model. Stein's model is a phenomenological representation of very complex electric and biochemical processes. We demonstrate its properties in terms of trajectories and stationary distribution. The subthreshold behavior of the membrane potential of a neuron cell is described in this model by a hybrid Markov process of continuous variable *V*(*t*) with jumps of constant intensity *λ *and constant amplitude in the continuous variable. Although in the original Stein model there are jumps of positive and negative sign, corresponding to excitatory and inhibitory synaptic activations, here we consider only positive sign jumps. If *t *is the moment of jump, then *V*(*t*^+^) - *V*(*t*^-^) = *a*, where *a *> 0 is the amplitude. Between two jumps *V *decays according to  where *α *is a constant.

The generator of such process is:



The steady probability distribution *p*(*x*) for such a process satisfies the following delay differential equation:

(25)

and *p*(*x*) = 0, for all *x *< 0.

**Figure 2:**
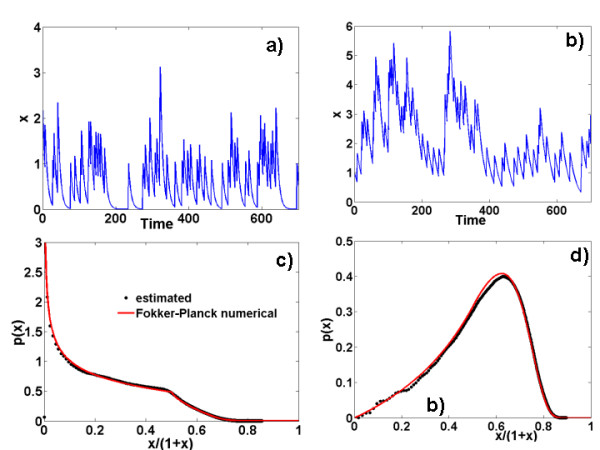
**Stein's model with excitatory synaptic activation (also bursting bacterium promoter with constant number of proteins produced per burst), an example of PDP with breakage**. Trajectories for a) *a *= 1, *α *= 0.15, *λ *= 0.1, b) *a *= 1, *α *= 0.05, *λ *= 0.1. Steady probability distributions c) same parameters as a); d) same parameters as b).

Analytical solutions are not known for this delay differential equation. In the Additional File 1 we describe the numerical scheme to calculate the steady probability distribution. The comparison between the simulated and calculated distribution is shown in Figure 2c-d. In Figure 2a-b we show trajectories of the system. Stein's model could also apply (even better, because its main defects with respect to neurons, such as the absence of a refractory period, is not a problem for genes) to gene networks. Generalizations of this model, allowing for continuous distributions of the jumps, could be used as an archetype of intermittent activity of a promoter.

Hybrid models with breakage can result as singular limits of hybrid models with switching as discussed in the Methods section *Hybrid approximations with singular switching*. The typical case is a bacterium operon. This model can be found in many places in literature. A detailed molecular example [63,64] will be studied as our last example (repressed operator site in a bacterium). A simpler version of this model is proposed by [65]. It consists of the following reactions:



The first reaction is zero order, all the other reactions are first order. The parameters satisfy *k*_1 _= *ak*_4_, *k*_2 _= *bk*_3_, *k*_4 _= *ϵk*_3_. We consider that *b *>> 1, *ϵ *<< 1.

From the aspect of the trajectories (these show bursting), the authors of [65] hypothetize that a PDP approximation with breakage is the natural simplification and solve the corresponding stationary hybrid Fokker-Planck equation for the protein component. We show here that the hybrid Fokker-Planck equation given in [65] can be found as an application of our approach.

First, we notice that a PDP limit is applicable to the Markov jump process. The species partition is *X*_*D *_= *mRNA*, *X*_*C *_= *Protein*. The mRNA component follows a birth and death process with birth intensity *k*_1 _and death intensity (*k*_3 _+ *k*_2_) *mRNA*. Using the master equation for birth and death processes [42] and considering that *k*_1_/(*k*_3 _+ *k*_2_) = *aϵ*/(1 + *b*) << 1, it follows that the probability to have zero or one molecule of mRNA is close to one (the probability to have two or more molecules is negligible).

This justifies that mRNA is a discrete species. The partition of the reactions is  = {*R*_1_, *R*_3_},  = {*R*_2_},  = {*R*_4_}. The timescale of the continuous variables is *τ *= (*k*_4_)^-1^. *V*_2 _= *k*_4 _*X *>> *τ*, where *X *is the number of proteins, provided that *X *>> 1. This condition, allowing application of the PDP approximation, is satisfied because *b *>> 1 (the significance of *b *is the average number of proteins produced in a bursting event).

Let us show that we have singular switching, equivalent to breakage. The reaction *R*_2 _is a super-reaction of the type  because *V*_2 _= *k*_2 _= *b*(*ϵτ*)^-1 ^The discrete variable *mRNA *can be considered to remain zero almost all the time, except during the negligible duration of the bursts when *mRNA *= 1. The reaction  corresponds to the transition 0 → 1, while the reaction  corresponds to the transition 1 → 0 of the discrete component *mRNA*. The intensity of the reaction *R*_3 _satisfies *V*_3 _= (*ϵτ*)^-1^. Thus, the mean duration of the burst is *ϵτ*. According to the section Methods, we are in the case of a PDP with singular switching. The evolution of the unique continuous variable *x *(protein concentration) is well approximated by the following hybrid generator (obtained after a simple change of the integration variable in Eq.(23)):



which shows that *b *is the mean number of proteins produced in a burst (mean size of the breakage).

We can notice a continuous distribution of the breakage size (exponential distribution), situation different from the Stein model. The Fokker-Planck equation can be solved in this case. The steady distribution of the continuous variable *x *is the Gamma distribution [65].

### First complex example: *λ*-phage toggle switch

In this section we revisit a classical example of toggle molecular switch. The model of cro-repressor (*cI*) switch in *λ*-phage, a temperate bacteriophage of Escherichia coli, was investigated by many authors [66-69].

The life cycle of phages has two alternative pathways, lysogenic when the phage duplicates synchronously with the host genome and lytic when the phage produces large amounts of its own mRNA. The switch between these two pathways is controlled by the level of *cI *protein. The lysogenic pathway corresponds to large levels of *cI*, while the lytic pathway corresponds to low levels of *cI*. A *cI *dimer may bind to any of the three operators: OR1, OR2, and OR3, in this order. By cooperativity, OR1 and OR2 are almost simultaneously occupied by *cI*. The third site OR3 will be occupied only when the *cI *concentration is high enough. A simpler model, in which OR1 is absent, may be considered [68]. Let *cI*, *cI*_2 _and *D *denote the repressor, repressor dimer, and DNA promoter site. The dynamics of the operator sites is described by the following reaction system:



where the *DcI*_2 _and  complexes denote binding to the OR2 or OR3 sites, respectively binding to both sites.

The other reactions concern production, degradation of molecules *cI*, *cI*_2_:



where *n *is the number of proteins per *mRNA *transcript.

The state vector is *X *= (*X*_*D*_, *X*_*C*_) where *X*_*D *_= {*D*, *DcI*_2_, , *DcI*_2_*cI*_2_}, *X*_*C *_= {*cI*, *cI*_2_}. Note that the choice of discrete variables is dictated here, like in our first example, by the conservation law *D *+ *DcI*_2 _+  + *DcI*_2_*cI*_2 _= *D*_0 _that restricts the numbers of *D*, *DcI*_2_, , *DcI*_2_*cI*_2 _to small values.

The partition of the species induces the following partition of the reactions:



The interesting property of the *λ*-phage model is its bistability. The naive calculation to find steady states uses quasi-stationarity of the deterministic dynamics. Then, we can use the equilibrium equations for reactions *R*_*i*_, *i *∈ [1,4], the steady state equation for *cI *and the conservation law to compute the concentration *x *of *cI *at steady-state. *x *= 0 is one of the steady state, namely the lytic attractor. It also corresponds to an absorbing state of the Markov process (the process always stops when it reaches this state). The lytic state can be rendered non-absorbing by including, like in [68], a small basal production rate of *cI *protein in the model. However, this is not important for the illustration of our approach. If *k*_2_/*k*_*m*2 _= *k*_3_/*k*_*m*3 _it follows that steady states (other than the lytic state) must satisfy:

(26)

where .

Let us notice that failure of naive quasi-stationarity calculations was demonstrated for nonlinear subsystems [37]. However, these calculations can be justified here by linear cycle averaging (the promoter sub-mechanism reactions *R*_*i*_, *i *∈ [2,4] are all pseudo-monomolecular).

Bistability occurs when the quartic equation (26) has real roots, which is the case when *σ *is small and *α *is big. More precisely, the model is bistable if . Like in [68] we have used *σ *= 5. In order to ensure bistability we have chosen *α *= 7.

**Figure 3:**
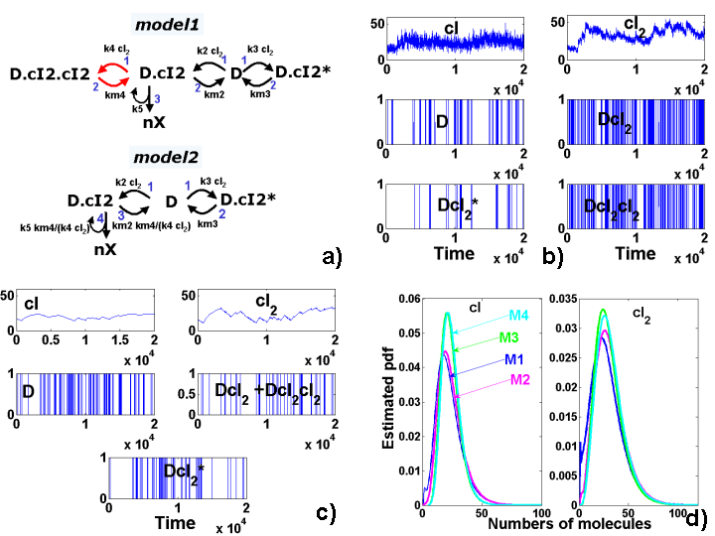
**Lambda-phage model, an example of averaged piecewise-deterministic process**. a) The cycle to be averaged is in red. Model 1 is the unreduced model, Model 2 is obtained from Model 1 by averaging. The integer labels represent orders of first order rate constants (1 represents the fastest reactions). b) SSA trajectory of the un-averaged model (Model 1). c) PDP trajectory of the averaged PDP (Model 4 which is the first order Kramers-Moyal approximation of Model 2). d) Comparison of estimated steady distribution for trajectories close to the lysogenic attractor (Models 3 and 4 are PDPs, obtained from Models 1 and 2, respectively).

The sub-mechanism *R*_*DC *_contains rapid unbroken cycles that need to be averaged before any further approximation of the process. These rapid cycles (see Figure 3a) correspond to rapid binding-unbinding of the dimer *cI*_2 _on the DNA. Three steps lead from the unreduced Model 1 to the averaged Model 2 (see also Figure 3a):

1.1 The cycle *DcI*_2_, *DcI*_2_*cI*_2 _is unbroken. It is glued to the node  whose total mass is equal to the mass of *DcI*_2 _and *DcI*_2_*cI*_2_.

1.2 The limiting step of the cycle is *k*_*lim *_= *k*_*m*4 _<<*k*_4 _*cI*_2_.

1.3 The branching reaction *DcI*_2 _→ *n X *+ *DcI*_2 _is replaced by  → *n X *+  of effective constant . The reaction *DcI*_2 _→ *D *+ *cI*_2 _is replaced by  → *D *+ *cI*_2 _with the reaction constant .

After averaging, two more cycles remain in the resulting Model 2. However, the rates of the remaining reactions *D *+ *DcI*_2 _→ *DcI*_2 _and *D *+ *DcI*_2 _→  are equivalent, which does not allow further application of our algorithm. Furthermore, the slow reactions  → *D *+ *cI*_2_, and  → *D *+ *cI*_2 _produce intermittence and should by no means be averaged.

The next approximation is a first order partial Kramers-Moyal expansion. The reactions in *R*_*C *_and the super-reaction *R*_5 _∈  contribute to the deterministic dynamics of the continous species defined by the following differential equations:

(27)

where *x*, *y *are the concentrations of *cI *and *cI*_2_, respectivelly.

The remaining reactions induce the jump mechanism.

The time evolution towards the lysogenic attractor is represented in Figure 3b for the un-reduced Model 1 and in Figure 3c for the PDP Model 4 (which is obtained by averaging and first order Kramers-Moyal expansion from Model 1). Steady probability distribution close to this attractor is represented in Figure 3d for all models in this study. We can notice the intermittent behavior of the *cI*, *cI*_2 _components that is well captured in the switching PDP approximations (Models 3 and 4).

### Second complex example: Stochastic bursting of a repressed bacterium operon

**Figure 4:**
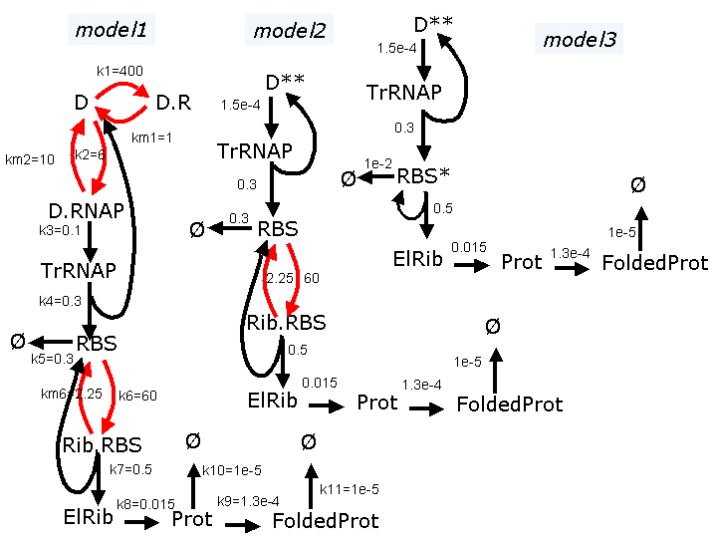
**Repressed bacterium operon**. The cycles to be averaged are in red. The numbers represent first order rate constants (including concentrations of buffered substrates where it is the case, here for *R *= 2500).

Under strong repression, protein production from a bacterium operator site undergoes stochastic bursting. A stochastic model for protein production in prokaryotes has been introduced in [63]. The behavior of the operator site under the regulation of a repressor molecule that prevents protein production has been considered in [64]. The corresponding model is represented in the Table 2 and in Figure 4.

**Table 2:** Set of reactions and parameters for the repressed bacterium operon model, from [64].

**Reaction**	**Parameters**
*Repressor Binding*	
	
1)	*k*1 = 10^8^*M*^-1^*s*^-1^, *km*1 = 1*s*^-1^
2)	*k*2 = 10^8^*M*^-1^*s*^-1^, *km*2 = 10*s*^-1^
3)	*k*3 = 0.1*s*^-1^
4)	*k*4 = 0.3*s*^-1^
	
*Translation*	
	
5)	*k*5 = 0.3*s*^-1^
6)	*k*6 = 10^8^*M*^-1^*s*^-1^, *km*6 = 2.25*s*^-1^
7)	*k*7 = 0.5*s*^-1^
8)	*k*8 = 0.015*s*^-1^
	
*Protein folding and decay*	
	
9)	*k*9 = (*ln*2/5400) *s*^-1^
10)	*k*10 = 10^-5^*s*^-1^
11)	*k*11 = 10^-5^*s*^-1^

In this model, the bacterium is considered to be in exponential growth phase, increasing size and dividing normally. Cell growth is simulated by a linear increase of the volume in time. During replication, the nuclear material doubles (variables D,D.R,DRNAP). At fission, the nuclear material is halved and all other components are divided among daughter cells according to a binomial distribution.

#### I. Species and reaction partition

Some of the components (*D*, *D.R*, *D.RN AP*, *Tr RN AP*) are confined to small numbers by the conservation law *D *+ *D.R *+ *D.RN AP *+ *Tr RN AP *= *const*.

Two more components *RBS *and *RibRBS *have small lifetimes ≈ 2*s *and can not accumulate to significant levels. Thus, the discrete variables are:



The remaining components are in large numbers and form the continuous variables:



We notice that Rib, RNAP and R are in relatively large numbers and practically constant, which justifies a first order reaction approximation for the mechanism *R*_*D*_. The sets of discrete species *D*, *D.R*, *D.RN AP *and *RBS*, *RibRBS *form rapid unbroken cycles and can be averaged.

#### II. Cycle averaging

The cycle averaging procedure can be applied three times:

1.1 The cycle *D*, *D.R *is unbroken. It is glued to the node D* whose total mass is equal to the mass of *D *and *D.R*.

1.2 The limiting step of the cycle is *k*_*lim *_= *k*_*m*1 _<<*k*_1_.

1.3 The branching reaction *D *→ *D.RN AP *is replaced by D* → *D.RN AP *of effective constant .

2.1 The cycle D*, *D.RN AP *is unbroken. It is glued to the node D** whose total mass is equal to the mass of *D *and *D.R *and *D.RN AP*.

2.2 We have  <<*k*_*m*2 _hence the limiting step of the cycle is .

2.3 The branching reaction *D.RN AP *→ *TrRN AP *is replaced by D** → *TrRN AP *of effective constant .

3.1 The cycle *RBS*, *Rib.RBS *is unbroken. It is glued to the node RBS* whose total mass is the one of *RBS *and of *Rib.RBS*.

3.2 The limiting step is *k*_*m*6 _<<*k*_6 _*Rib*.

3.3 The branching reaction *Rib.RBS *→ *ElRib *+ *RBS *is replaced by the reaction RBS* → *ElRib *+ RBS* of effective constant *k7' *= *k*7.

3.4 The branching reaction *RBS *→ ∅ is replaced by the reaction RBS* → ∅ of effective constant .

Notice that a loss of accuracy should be expected from the application of the third averaging step. The separation of the branching and cycling reaction rate constants is not that good. Indeed, *k*_7_/*k*_*m*6 _≈ 0.22 while in theory we need *k*_7_/*k*_*m*6 _<< 1.

#### III. Identification of super-reactions

Notice that after cycle averaging, a low intensity reaction D** → *TRN AP *results, producing intermittency (bursting). The reduced mechanism is represented in Figure 4b. The discrete/continuous partition of the species in the reduced mechanism is inherited from the initial model.

In the reduced mechanism, the reaction RBS* → RBS* + *ElRib *is very quick, even quicker than the continuous reactions *ElRib *→ *Prot*, *Prot *→ *FoldedProt*, *FoldedProt *→ ∅, therefore it is a super-reaction of the type .

#### IV. Hybrid approximation

First order Kramers-Moyal expansion applied to the averaged Markov jump process leads to a PDP with switching.

The continuous variables obey to the following differential equations:

(28)

**Figure 5:**
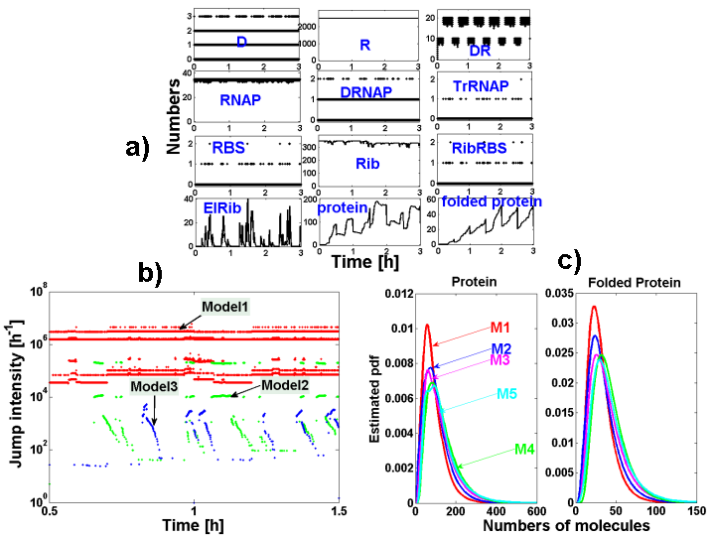
**Repressed bacterium operon**. a) Trajectories for *R *= 2500. b) Jump intensities for three SSA versions of the repressed bacterium operon. Model 1 (without averaging), Model 2 (averaging of *D*, *D.R*, *D.RN AP*), Model 3 (averaging of *D*, *D.R*, *D.RN AP*, *RBS*, *Rib.RBS*). c) Steady probability distributions for the protein and folded protein obtained with the five versions of the model, for *R *= 2500. *M*3 and *M*4 are PDP obtained from *M*2 and *M*3, respectively.

The remaining three discrete components (D**, *TrRN AP*, RBS*) form a Markov jump process. Inside the reaction mechanism there is a rapid chain leading from *TrRN AP *to RBS* and to *ElRib *production which is fed by a extremely slow reaction producing *TrRN AP*. Thus RBS* presents unfrequent bursts of activity leading to rapid production of *ElRib*. The increasing part of the burst does not last long, because the discrete component RBS* rapidly switches back to zero by the reaction RBS* → ∅. Thus, in the continuous variables, after a steep (deterministic) increase of ElRib one observes a slower decrease. We have the case of a PDP with switching, where the steep increase event could be assimilated to a breakage (see Figure 5a). However, the timescales of the decreasing and increasing parts of the peak are not well separated. Thus, the breakage approximation could be used only to obtain qualitative results.

To summarize, we have considered five models: the unreduced Markov jump model, two averaged reduced jump Markov models (the second model obtained after averaging steps 1 and 2, the third model after application of all three steps) and two PDP models with switching obtained from each of the previous two averaged jump Markov models. The three jump Markov models are simulated using the Gillespie algorithm, and the two hybrid models are simulated with the PDP algorithm.

Of course, the process is Markovian only between two cell cycle events that are under external command. Considering the external command, the process is semi-Markovian [29,70]. We could restore the Markovian framework, by considering a cell cycle variable that has periodic autonomous dynamics and triggers the transitions between the cell cycle stages.

In order to compare the performance of the models (in terms of time complexity) we have represented the total jump intensities for the first three models (exact SSA, and the two averaged models) as functions of time on a trajectory. The model that demands the least computer time is the one with the smallest jump intensity. In Figure 5b, we notice a decrease of several orders of magnitudes of the total intensity from the exact SSA model to the models obtained after the second and third averaging steps.

We can not use the same method to estimate the computation time of the PDP algorithm. Indeed, although it is true generally that the computation time increases with the number of discrete transitions, the number of operations to compute the deterministic parts depends on the deterministic solver. In order to reduce the number of operations per time point we have favored one-step schemes (although this is not important for very large systems, where the main difficulty is represented by the computation of the flow function). We also favored implicit stiff solvers that can function with large time steps, reducing computation time. All the calculations were performed by using ode23s solver of MATLAB (this is a one-step implicit stiff solver using Rosenbrock formula of order 2). A comparison of the times to generate a 20 cycles long trajectories with the different methods is given in Table 3. This table is rather illustrative of the advantages of various approximations. Averaging allows a tremendous reduction of the execution time at least 50 times for weak repression and 10^4 ^times for strong repression (when stochastic effects are strongest). The Kramers-Moyal expansion leading to PDPs produce an extra 2-fold decrease of the execution time, but this is true only for weak repression and after averaging of all rapid cycles. The PDP model M4 with incomplete averaging of fast cycles has a very bad performance, that can be even worse than the non-averaged SSA. This can be explained by the strong stiff character of the deterministic dynamics with steep ElRib peaks.

**Table 3:** Execution times for the repressed bacterium operon model.

***R***	**M1 - SSA**	**M2 - averaging + SSA**	**M3 - averaging + SSA**	**M4 - PDP ode23s**	**M5 - PDP ode23s**
50	7.4 10^3^	2.9 10^3^	1.4 10^2^	1.1 10^4^	6.7 10^1^
100	6.8 10^3^	6.9 10^2^	3.5 10^1^	3.7 10^3^	3.6 10^1^
200	5.4 10^3^	1.7 10^2^	1.3 10^1^	1.6 10^3^	1.9 10^1^
300	4.9 10^3^	8.1 10^1^	7.6	8.0 10^2^	1.4 10^1^
2500	3.7 10^3^	2.3	5.2 10^-1^	1.0 10^2^	4.1
5000	3.6 10^3^	8 10^-1^	1.3 10^-1^	4.7 10^1^	2.9
10000	3.6 10^3^	1 10^-1^	2.1 10^-2^	3.1 10^1^	2.0

The time, expressed in [s] correspond to generating a 20 division cycles long trajectory (3.6 10^4^s biological time) for models M1-M5. The values represent log-averages for 10 launches. Model M4 is obtained from M2 and model M5 is obtained from M3, via the Kramers-Moyal expansion.

To test the accuracies of various approximations we have computed the steady probability distribution of the protein and of the folded protein using trajectories generated by the five models. The distribution obtained by SSA for the un-reduced model is used as the "exact" reference. The observed errors are the consequences of less good separation between systems constants. For instance, in Figure 4, *k*_7 _= 0.5 and *k*_*m*6 _= 2.25, while in theory we need *k*_7 _<<*k*_*m*6_. However, the approximate models are qualitatively correct. All models render correctly the bursting behavior of the system.

Another advantage of a simplified model is the reduced number of parameters. The full SSA model has 14 parameters. After the first two averaging steps only 9 parameters remain, and after the three averaging steps only 7. The PDPs have the same number of parameters as the corresponding averaged Markov jump processes, namely 9 and 7 parameters. Furthermore, the parameters of the simplified models are monomials of parameters of the unreduced model. This can be used for sensitivity analysis. After identification of the monomials that are critical for a given property the backtracked in uence of the initial parameters is given by the power of the corresponding parameter in the critical monomial. The details of the method can be found in [36].

## Conclusion

We have presented, in a unified framework, various hybrid simplifications of stochastic biochemical models. These simplifications are based on partial Kramers-Moyal expansions and on averaging.

The use of simplified models in stochastic studies of cellular processes has several advantages.

The first advantage of simplified models is the reduction of computational time. We have shown that cycle averaging leads to the most drastic reduction of the computation time. This averaging algorithm, that can be used independently of the Kramers-Moyal expansion, represents a general preconditioning method for both stochastic and deterministic simulations. In stochastic studies, it reduces the number of discrete events to be simulated. In deterministic studies, it produces less stiff systems. The preconditioned models can be the starting point for other reduction methods.

Mathematically, our simplified models are weak approximations of the fully detailed jump Markov processes. This means that all statistical properties of the trajectories of the full model including steady distributions, transition times, etc. should be rendered without significant loss of accuracy by the simplified models. Of course, after the reduction procedure, some variables and reactions may disappear and some resulting parameters are synthetic. Because the correspondence with the original variables and parameters is known, it is always possible to go back to the details of the full model. In particular, the identification of the critical parameters of the reduced model allows to backtrack the critical parameters of the full model. The Kramers-Moyal expansion, leading to hybrid simplifications, produced only a moderate decrease of the computation time. This limitation was partly due to our choice of low to medium size models with rather small numbers of molecules and with simple dynamics of the continuous variables. More obvious computational advantage can be obtained for more complex models. Particularly, this could be the case for models with oscillating dynamics of the continuous variables, such as molecular clocks.

The second advantage of simplified models lies in the understanding that these bring with respect to the stochastic properties of the system. Averaging and hybrid simplifications unravel the origin of noise in multiscale biochemical systems. Noise is generated at the microscopic level of discrete variables and transferred to the mesoscopic level of the the continuous variables. In this transfer, the topology of the reaction network plays a role, but also other properties are important such as the hierarchy of timescales of the system. Our simplification algorithm explains how and when hierarchies of cycles lead to intermittence and noise in gene networks. In many cases, important properties such as the stationary distribution, noise amplitude, waiting times between successive bursts can be analytically calculated for the hybrid simplifications.

We have discussed several types of hybrid approximations: piecewise deterministic and hybrid diffusions with switching, with breakage, and singular switching that can be assimilated to breakage. Taken together, these results offer a rather complete panorama of various intermittence phenomena in biochemical systems.

## Authors' contributions

OR proposed the methodology to obtain hybrid simplifications. AC and OR chose the examples and implemented the algorithms. AC, AD and OR contributed to the mathematical aspects of the methods part. All authors drafted, read and approved the final manuscript.

## Supplementary Material

Additional file 1 **Additional material**. This file contains mathematical proofs of some results concerning averaging and singular switching. It also contains a description of the methods to estimate steady probability density function for PDPs. Click here for file
