# Supplementary material for: Hybrid stochastic simplifications for multiscale gene networks
Source: BMC Syst Biol. 2009 Sep 7;3:89. doi: 10.1186/1752-0509-3-89 (PMC2761401; doi:10.1186/1752-0509-3-89)
Supplement: Additional file 1 — Additional material. This file contains mathematical proofs of some results concerning averaging and singular switching. It also contains a description of the methods to estimate steady probability density function for PDPs. [file 1752-0509-3-89-S1.pdf]

Additional material for the paper Hybrid stochastic simplifications for multiscale gene networks

Alina Crudu [1], Arnaud Debussche [2,3] , Ovidiu Radulescu *[1,3]

[1] IRMAR UMR CNRS 6625 Université de Rennes1, Campus de Beaulieu, 35042 Rennes, France
[2] ENS Cachan, Antenne de Bretagne Avenue Robert Schumann, 35170, BRUZ, France
[3] INRIA-IRISA, Campus de Beaulieu, 35042 Rennes, France

Email: Alina Crudu - alina.crudu@univ-rennes1.fr ; Arnaud Debussche - arnaud.debussche@bretagne.ens-cachan.fr; Ovidiu Radulescu *- ovidiu.radulescu@univ-rennes1.fr;

*Corresponding author

1 Numerical scheme to calculate the steady probability distribution for Stein's model

The infinitesimal generator reads

$$Af(x) = -\alpha x f'(x) + \lambda(f(x+a) - f(x)).$$

The adjoint of the infinitesimal generator can be found from the relation $\int_0^\infty p(x)(Af(x))dx = \int_0^\infty (A^* p(x))f(x)dx$ with the condition $p(x) = 0$, for all $x \leq 0$. It follows that

$$A^* p(x) = \alpha x p'(x) + \alpha p(x) + \lambda(p(x-a) - p(x)).$$

The steady probability distribution is the solution of the following differential equation with delays

$$A^* p(x) = 0.$$

with $p(x) = 0$, for all $x \leq 0$.

In order to solve this equation numerically on $[0, \infty[$ we reduce the problem to an equivalent one defined on the bounded set $[0, 1]$ and use the method of finite differences to approximate derivatives.

First, we use the substitution

$$p(x) = g(z), \quad z = \frac{x}{1+x},$$

that transforms the domain of the problem from $[0, \infty[$ to the interval $[0, 1[$ and the equation to solve becomes

$$\alpha g(z) + \alpha z(1-z)g'(z) + \lambda \left[g\left(\frac{z - a(1-z)}{1 - a(1-z)}\right) - g(z) \right] = 0,$$

for all $z \in]0, 1[$.

Thus, we use an equidistant discretisation of $[0, 1]$ ($z_i = ip, p = 1/n, n \in \mathbb{N}^*$).

We get the following system

$$\alpha i(1-ip)\left[g((i+1)p) - g(ip)\right] + \alpha g(ip) + \lambda \left[g\left(\frac{i - \frac{a}{p}(1-ip)}{1 - a(1-ip)} p\right) - g(ip) \right] = 0,$$

for all $0 \leq i \leq n$-1. We let $X_i = g(ip)$ and get the following linear system

$$[-\alpha i(1-ip) + a - \lambda]X_i + \alpha i(1-ip)X_{i+1} + \lambda X_j = 0,$$

for all $i \leq n-1$ (where j is chosen to be the positive integer closest to $\frac{i - \frac{a}{p}(1-ip)}{1-a(1-ip)}$).

2 The numerical method to estimate stationary distributions

The stationary distributions of various pure jump and piece-wise deterministic processes were estimated by a Monte Carlo method. We consider a fixed time horizon $TMAX$. First, a trajectory is simulated using the PDP algorithm (see main text). Then, the initial part of the trajectory between 0 and $TMIN$ is discarded. The choice of the equilibration time $TMIN$ should be larger than the slowest relaxation time of the process. We also assume ergodicity. This hypothesis guarantees uniqueness of the steady probability distribution and also allows us to estimate the steady probability distribution by using the part of the trajectory between $[TMIN, TMAX]$.

The truncated trajectory of a pure jump process is a series of times (jump instants) $t0 = TMIN < t_2 < \ldots < t_N = TMAX$ and a corresponding series of states $x_0, \ldots, x_N$. Considering that the process is constant between t_i and t_{i+1}, the steady probability density function can be estimated as a histogram of the values x_i with weights $t_{i+1} - t_i$.

For piece-wise deterministic processes, the assumption that the process is constant between two successive jump instants is no longer satisfied. In this case, the interval between two jump instants is sampled by the deterministic solver. Considering that the time step $\delta t = t_{i+1} - t_i$ is small enough, a linear approximation is suitable for the histogram, corresponding to the trapezium rule: x_i should be replaced by $(x_i + x_{i+1})/2$.

Sometimes, we choose to resample the deterministic pieces of the trajectories, using uniform discretisation and spline interpolation.

3 Justification of averaging results

In order to obtain averaged hybrid approximations from the master equation we generally need two parameters. A large parameter $\mathscr{V}$ (representing volume) is used to rescale large molecule numbers as in Kramers-Moyal or Ω expansions. A small parameter ϵ (representing ratio of fast to slow timescales) is used to separate fast dynamics within cycles and slow dynamics between cycles. In the most general case, Kramers-Moyal and averaging can be performed in one step starting with the master equation and developing with respect to $1/\mathscr{V}$ and ϵ. This is a singular perturbation problem because the dynamics at $\epsilon > 0$, $1/\mathscr{V} > 0$ is not an uniform approximation of the dynamics at $\epsilon = 0$, $1/\mathscr{V} = 0$. Arbitrarily large differences between the two dynamics occur if we wait long enough. For instance, weakly coupled cycles have a different long time dynamics and reach a different steady state than totally uncoupled cycles.
We can consider the following cases:

Case 1 : Fast discrete cycles producing continuous species. There are fast super-reactions of the type 1 that act on discrete species $\gamma_i^D \neq 0$ for some $i \in \mathscr{S}_1$. In this case there is only one small parameter $\eta = 1/\mathscr{V}$.

Case 2 : Fast discrete cycles. In this case there are some fast transitions between discrete species. The small parameter is ϵ.

Case 3 : A combination of the above two cases. There are two small parameters, η and ϵ.

We discuss only the first two cases.

Case 1 The conditions defining the Case 1 mean that some rapid cycles change in the same time discrete and continuous variables. The discrete species change within a finite set of values, and remain in small numbers. The continuous species can be produced continuously, in large numbers. This case is similar to the QSSA SPA-Ω expansion in [1]. Our discrete species are the quasi-stationary species in [1]. The difference is that here we consider rapid cycling (implying production and consumption) of discrete species, while in [1] only rapid consumption is considered (meaning that $\gamma_i^D < 0$ for $i \in \mathscr{S}_1$. To simplify, we consider that all reactions in $\mathscr{R}_{DC}$ are of this type ($\mathscr{R}_{DC} = \mathscr{S}_1$ and $\gamma_i^D \neq 0$ for all reactions in $\mathscr{R}_{DC}$).

Let $x_c = \eta X_c$ be the concentrations of the continuous species. Let X_D^2 be the discrete species that are affected by super-reactions of the type 1 and X_D^1 the remaining discrete species.

We start from the following master equation:

$$\begin{aligned}
\frac{\partial p}{\partial t}(X_D^1, X_D^2, x_c, t) = &\sum_{i \in \mathscr{R}_C} \eta^{-1} v_i(x_c - \eta\gamma_i^C) p(X_D^1, X_D^2, x_c - \eta\gamma_i^C, t) - \eta^{-1} p(X_D^1, X_D^2, x_c, t) \sum_{i \in \mathscr{V}_C} v_i(x_c) + \\
+ &\sum_{i \in \mathscr{R}_D} V_i(X_D^1 - \gamma_i^{D,1}, X_D^2 - \gamma_i^{D,2}) p(X_D^1 - \gamma_i^{D,1}, X_D^2 - \gamma_i^{D,2}, x_c, t) - p(X_D^1, X_D^2, x_c, t) \sum_{i \in \mathscr{R}_D} V_i(X_D^1, X_D^2) \\
+ &\sum_{i \in \mathscr{R}_{DC}} \eta^{-1} v_i(X_D^1, X_D^2 - \gamma_i^{D,2}, x_c - \eta\gamma_i^C) p(X_D^1, X_D^2 - \gamma_i^{D,2}, x_c - \eta\gamma_i^C, t) - \\
- &\eta^{-1} p(X_D^1, X_D^2, x_c, t) \sum_{i \in \mathscr{R}_{DC}} v_i(X_D^1, X_D^2, x_c)
\end{aligned} \tag{1}$$

We develop p as a power series in η:

$$p(X_D^1, X_D^2, x_c, t) = p_0(X_D^1, X_D^2, x_c, t) + \eta p_1(X_D^1, X_D^2, x_c, t) + \dots$$

This series will provide the large time solution of the master equation. Using a Taylor expansion of (1) we obtain at various orders:

At order η^{-1}

$$\sum_{i \in \mathscr{R}_{DC}} v_i(X_D^1, X_D^2 - \gamma_i^{D,2}, x_c) p_0(X_D^1, X_D^2 - \gamma_i^{D,2}, x_c, t) - p_0(X_D^1, X_D^2, x_c, t) \sum_{i \in \mathscr{R}_{DC}} v_i(X_D^1, X_D^2, x_c) = 0$$

This means that at X_D^1, x_c, t fixed, p_0 is the steady-state distribution of the process defined by the fast reactions $\mathscr{R}_{DC}$. Now, we consider that this process is ergodic, meaning that starting from any state we can reach any other state in finite time. The reaction mechanism $\mathscr{R}_{DC}$ should be made of one or several interconnected cycles. This condition ensures the uniqueness of the steady state distribution and we can write that:

$$p_0(X_D^1, X_D^2, x_c, t) = \psi(X_D^1, x_c, t) \rho(X_D^2) \tag{2}$$

where $\rho(X_D^2)$ is the unique steady state distribution of the fast process and $\psi(X_D^1, x_c, t)$ is the time-dependent distribution of the remaining variables.

At order η^0

$$\frac{\partial p_0}{\partial t} = -\nabla_{x_c}.[\chi_c(x_c)p_0]+$$
$$+\sum_{i\in\mathscr{R}_D} V_i(X_D^1-\gamma_i^{D,1}, X_D^2-\gamma_i^{D,2})p_0(X_D^1-\gamma_i^{D,1}, X_D^2-\gamma_i^{D,2}, x_c, t) - p_0(X_D^1, X_D^2, x_c, t)\sum_{i\in\mathscr{R}_D} V_i(X_D^1, X_D^2)$$
$$-\nabla_{x_c}.[\sum_{i\in\mathscr{R}_{DC}} v_i(X_D^1, X_D^2-\gamma_i^{D,2}, x_c)\gamma_i^C p(X_D^1, X_D^2-\gamma_i^{D,2}, x_c, t)]+$$
$$+\sum_{i\in\mathscr{R}_{DC}} v_i(X_D^1, X_D^2-\gamma_i^{D,2}, x_c)p_1(X_D^1, X_D^2-\gamma_i^{D,2}, x_c, t) - p_1(X_D^1, X_D^2, x_c, t)\sum_{i\in\mathscr{R}_{DC}} v_i(X_D^1, X_D^2, x_c) \quad (3)$$

where $\chi_c(x_c) = \sum_{i\in\mathscr{R}_C} v_i\gamma_i^C$.

By using (2) and by summing (3) with respect to all possible values of X_D^2 we obtain:

$$\frac{\partial \psi}{\partial t} = -\nabla_{x_c}.[(\chi_c(x_c)+\bar{\chi}_{DC}(X_D^1, x_c))\psi(X_D^1, x_c, t)]+$$
$$+\sum_{i\in\mathscr{R}_D} \bar{V}_i(X_D^1-\gamma_i^{D,1})\psi(X_D^1-\gamma_i^{D,1}, x_c, t) - \psi(X_D^1, x_c, t)\sum_{i\in\mathscr{R}_D} \bar{V}_i(X_D^1) \quad (4)$$

where $\bar{V}_i(X_D^1) = \sum_{X_D^2} V_i(X_D^1, X_D^2)\rho(X_D^2)$, $\bar{\chi}_{DC}(X_D^1, x_c) = \sum_{X_D^2}\sum_{i\in\mathscr{R}_{DC}} v_i(X_D^1, X_D^2, x_c)\gamma_i^C$ are averaged rates.

Eq.(4) shows that the zeroth order approximation is an averaged PDP.

Case 2 We do not need to distinguish here between discrete and continuous species. The reader could imagine the situation when there are only discrete species. We consider that there are fast reactions $\mathscr{R}_2$ and slow reactions $\mathscr{R}_1$. We consider that $V_i = \epsilon^{-1}v_i$ for $i \in \mathscr{R}_2$. Like for Case 2 we consider two types of species. X_D^2 are the species that are affected by fast reactions and X_D^1 the remaining species.

This case can be treated exactly in the same way as the preceding case. We start from the following master equation:

$$\frac{\partial p}{\partial t}(X_D^1, X_D^2, t) = \sum_{i\in\mathscr{R}_1} V_i(X_D^1-\gamma_i^{D,1}, X_D^2-\gamma_i^{D,2})p(X_D^1-\gamma_i^{D,1}, X_D^2-\gamma_i^{D,2}, t)-$$
$$-p(X_D^1, X_D^2, t)\sum_{i\in\mathscr{R}_1} V_i(X_D^1, X_D^2) + \sum_{i\in\mathscr{R}_2}\epsilon^{-1}v_i(X_D^1-\gamma_i^{D,1}, X_D^2-\gamma_i^{D,2})p(X_D^1-\gamma_i^{D,1}, X_D^2-\gamma_i^{D,2}, t)- \quad (5)$$
$$-p(X_D^1, X_D^2, t)\sum_{i\in\mathscr{R}_2}\epsilon^{-1}v_i(X_D^1, X_D^2)$$

With the same assumption on the ergodicity of the process defined by fast reactions, we have $p = \psi(X_D^1, t)\rho(X_D^2) + \epsilon p_1(X_D^1, X_D^2, t)$. In the zero-th approximation, $\rho(X_D^2)$ is the steady state distribution of the fast mechanism and $\psi(X_D^1, t)$ represents the dynamics of an averaged mechanism:

$$\frac{\partial \psi}{\partial t}(X_D^1, t) = \sum_{i \in \mathscr{R}_1} \bar{V}_i(X_D^1 - \gamma_i^{D,1})\psi(X_D^1 - \gamma_i^{D,1}, t) - \psi(X_D^1, t) \sum_{i \in \mathscr{R}_1} \bar{V}_i(X_D^1) \quad (6)$$

where $\bar{V}_i(X_D^1) = \sum_{X_D^2} V_i(X_D^1, X_D^2)\rho(X_D^2)$ are averaged rates.

When the mechanism $\mathscr{R}_2$ is made of a single cycle $A_1^2 \to A_2^2 \to ...A_m^2 \to A_1^2$, the steady state distribution can be easily calculated. This distribution is parametrised by the value of the total mass of the cycle $N = \sum_{i=1}^m X_i^2$, which is a slow variable. Consider total separation of the reaction constants of the cycle and that last step is limiting. Then, mass is concentrated with probability $1 - \sum_{i \neq m} k_{\lim}/k_i$ close to one to the beginning of the limiting step and with small probabilities $k_{\lim}/k_i$ at the beginning of step $i \neq m$. The average of X_j^2 with respect to the steady distribution ρ is $\bar{X}_j^2 = N k_{\lim}/k_j$. Then the average rate of the reaction $A_j^2 \to B$ of reaction constant k where B is exterior to the cycle is $\bar{V}_j = k\bar{X}_j^2 = N k k_{\lim}/k_j$.

4 Justification of the singular switching results

Consider the following PDP with singular switching. The flow function is:

$$\chi(X_D, x) = \chi_0(x) + \frac{1}{\epsilon}\chi_1(X_D, x)$$

where

$$\chi_0(x) = \sum_{i \in \mathscr{R}_C} \gamma_i^C v_i(x),\ \chi_1(X_D, x) = \sum_{i \in \mathscr{S}_3} \gamma_i^C v_i(X_D, x),$$

and the jump intensity is

$$\lambda(X_D, x) = \sum_{i \in \mathscr{R}_D \setminus \mathscr{R}_D^-} V_i(X_D) + \frac{1}{\epsilon} \sum_{j \in \mathscr{R}_D^-} v_j(X_D).$$

We shall obtain the limit process by two methods. The first method employs a scaling argument, and the second method employs the expansion of the hybrid Fokker-Planck equation.

The state of the system is (x, X_D, X_D') where x are continuous species, X_D are discrete species, and X_D' are discrete species substrates of of rapid reactions of the type $\mathscr{S}_3$ that are responsible for the breakage. To simplify the calculations we consider that variables X_D' take only two values $X_D' \in \{0,1\}$. If $X_D' = 1$ species x are rapidly produced by reactions $\mathscr{S}_3$, and if $X_D' = 0$ production stops. Once in the state $X_D' = 1$, the system rapidly switches back to $X_D' = 0$ by fast reactions $\mathscr{R}_D^-$.

Scaling argument Let us consider the process in a state $(X_D, X'_D = 0, x)$. With intensity $V_i(X_D)$ it jumps to the state $(X_D + \gamma_i^D, X'_D = 1, x)$, where it stays only for a very short time. In this state, the continuous variables are submitted to the fast dynamics:

$$\frac{dx_\epsilon}{dt} = \frac{1}{\epsilon}\chi_1(X_D + \gamma_i^D, x_\epsilon),\ x_\epsilon(0) = x$$

during the short random time τ_ϵ that satisfies

$$P[\tau_\epsilon > t] = \exp[-\frac{1}{\epsilon}v_j(X_D + \gamma_i^D)t] \tag{7}$$

then jumps to the state $X_D + \gamma_i^D + \gamma_j^D$, $X'_D = 0$ by a reaction $j \in \mathscr{R}_D^-$.

Let $\Phi(s; x, X_D)$ be the solution of the equation

$$\frac{d\Phi}{ds} = \chi_1(\Phi, X_D), \quad \Phi(0) = x$$

Passing to short timescales $s = t/\epsilon$, we can easily show that the variation of the continuous variable starting from x and during the time τ_ϵ is:

$$\Delta x_\epsilon = x_\epsilon(\tau_\epsilon) - x = \Phi(\tilde{s}; x, X_D + \gamma_i^D) - x$$

where the random time $\tilde{s}$ satisfies

$$P[\tilde{s} > s] = \exp[-v_j(X_D + \gamma_i^D)s]$$

Keeping the random variable Δx_ϵ as jump (breakage) of the continuous variable and noticing from (7) that $\tau_\epsilon \to 0$ (almost surely), we obtain the following generator of the limit hybrid process:

$$\begin{aligned}\mathscr{A}f(X_D, x) = \chi_0(x).\nabla_x f + \sum_{i \in \mathscr{R}_D \setminus (\mathscr{R}_D^- \cup \mathscr{R}_D^+)} V_i(X_D)[f(X_D + \gamma_i, x) - f(X_D, x)] + \\ + \sum_{i \in \mathscr{R}_D^+} V_i(X_D) \sum_{j \in \mathscr{R}_D^-} \int_0^\infty [f(X_D + \gamma_i^D + \gamma_j^D, \Phi(s; x, X_D + \gamma_i^D)) - f(X_D, x)]\rho_{ij}(s)ds\end{aligned} \tag{8}$$

where $\rho_{ij}(s) = v_j(X_D + \gamma_i^D)\exp[-v_j(X_D + \gamma_i^D)s]$ is a probability density satisfying $\int_0^\infty \rho_{ij}(s)ds = 1$.

From the generator we can obtain the hybrid Fokker-Planck equation (using the adjoint of the generator). In the adjoint, we should replace trajectories starting from x to trajectories arriving in x, thus changing s

into $-s$ and renormalizing the density to take into account the phase space volume transformation. Thus, the hybrid Fokker-Planck equation reads:

$$\begin{aligned}
\frac{\partial p}{\partial t}(X_D, x, t) = & -\nabla_x.(\chi_0(x) p(X_D, x, t)) + \\
& + \sum_{i \in \mathscr{R}_D \setminus (\mathscr{R}_D^- \cup \mathscr{R}_D^+)} p(X_D - \gamma_i, x, t) V_i(X_D - \gamma_i) - p(X_D, x, t) \sum_{i \in \mathscr{R}_D \setminus (\mathscr{R}_D^- \cup \mathscr{R}_D^+)} \sum V_i(X_D) + \\
& + \sum_{i \in \mathscr{R}_D^+} \sum_{j \in \mathscr{R}_D^-} V_i(X_D - \gamma_i^D - \gamma_j^D) \int_0^\infty p(X_D - \gamma_i^D - \gamma_j^D, \phi(-s; x, X_D - \gamma_j^D), t) \rho_j'(s) ds - \\
& - p(X_D, x, t) \sum_{i \in \mathscr{R}_D^+} V_i(X_D)
\end{aligned} \tag{9}$$

where $\rho_j'(s) = v_j(X_D - \gamma_j^D) \exp[-\int_0^s (\lambda_-(X_D - \gamma_j^D) + \nabla_x . \chi_1(X_D - \gamma_j^D, \phi(-s'; x, X_D - \gamma_j^D))) ds']$.

Fokker-Planck equation argument To simplify formulae, we consider with no loss of generality that $\mathscr{R}_D = \mathscr{R}_D^- \cup \mathscr{R}_D^+$.

The hybrid Fokker-Planck equation for the singular switching process reads:

$$\begin{aligned}
\frac{\partial p}{\partial t}(X_D, 1, x, t) = & -\nabla_x.[p(X_D, 1, x, t)(\chi_0(x) + \frac{1}{\epsilon}\chi_1(X_D, x))] + \sum_{i \in \mathscr{R}_D^+} p(X_D - \gamma_i^D, 0, x, t) V_i(X_D - \gamma_i^D) - \\
& - \frac{1}{\epsilon}\lambda_-(X_D) p(X_D, 1, x, t) \\
\frac{\partial p}{\partial t}(X_D, 0, x, t) = & -\nabla_x.[(p(X_D, 0, x, t)\chi_0(x)] + \frac{1}{\epsilon} \sum_{j \in \mathscr{R}_D^-} p(X_D - \gamma_j^D, 1, x, t) v_j(X_D - \gamma_j^D, 1) - \\
& - \lambda_+(X_D) p(X_D, 0, x, t)
\end{aligned} \tag{10}$$

where

$\lambda_-(X_D) = \sum_{j \in \mathscr{R}_D^-} v_j$, $\lambda_+(X_D) = \sum_{j \in \mathscr{R}_D^-} V_j$.

Consider that $p(X_D, X_D', x, t) = p_0(X_D, X_D', x, t) + \epsilon p_1(X_D, X_D', x, t) + \dots$

Then we Taylor expand the Fokker-Planck equations (10) and we obtain:

At order ϵ^{-1}

$$p_0(X_D, 1, x, t) = 0$$

At order ϵ^0

$$
\begin{aligned}
&-\nabla_x[p_1(X_D,1,x,t)\chi_1(X_D,x)] + \sum_{i\in\mathscr{R}_D^+} p_0(X_D-\gamma_i^D,0,x,t)V_i(X_D-\gamma_i^D) - \lambda_-(X_D)p_1(X_D,1,x,t) = 0\\
&\frac{\partial p_0}{\partial t}(X_D,0,x,t) = -\nabla_x[p_0(X_D,0,x,t)\chi_0(x)] + \sum_{j\in\mathscr{R}_D^-} p_1(X_D-\gamma_j^D,1,x,t)v_j(X_D-\gamma_j^D,1)-\\
&-\lambda_+(X_D)p_0(X_D,0,x,t)
\end{aligned} \tag{11}
$$

From the first of the Eqs.11 we obtain

$$
\begin{aligned}
\frac{dp_1(*,1,\Phi(-s;x,**),t)}{ds} =& [\lambda_-(*) + \nabla_x.\chi_1(X_D,\Phi(-s;x,**))]p_1(*,1,\Phi(-s;x,**),t)-\\
&-\sum_{i\in\mathscr{R}_D^+} p_0(*-\gamma_i^D,0,\Phi(-s;x,**),t)V_i(*-\gamma_i^D)
\end{aligned} \tag{12}
$$

The linear differential equation (12) has the solution:

$$
\begin{aligned}
&p_1(*,1,x,t) = p_1(*,1,\Phi(0;x,**),t) =\\
&= \int_0^\infty \sum_{i\in\mathscr{R}_D^+} p_0(*-\gamma_i^D,0,\Phi(-s;x,**),t)V_i(*-\gamma_i^D)exp[-\int_0^s(\lambda_-(*)+\nabla.\chi_1(X_D,\Phi(-s';x,**)))ds']ds.
\end{aligned} \tag{13}
$$

By replacing (13) in the second of the Eqs.11 we obtain again Eq.(9).

References

1. Mastny E, Haseltine E, Rawlings J: **Two classes of quasi-steady-state model reductions for stochastic kinetics**. *The Journal of Chemical Physics* 2007, **127**:094106.
